# Fecal microbiota transplantation augments 5-fluorouracil efficacy in pancreatic cancer via gut microbiota modulation

**DOI:** 10.3389/fmicb.2025.1548027

**Published:** 2025-09-25

**Authors:** Rui Li, Yaoyuan Hu, Yixian Liu, Xiaodong Tan

**Affiliations:** ^1^Department of General Surgery, Shengjing Hospital of China Medical University, Shenyang, China; ^2^Liaoning University of Traditional Chinese Medicine, Shenyang, China

**Keywords:** fecal microbiota transplantation, 5-fluorouracil, pancreatic cancer, gut microbiota, short-chain fatty acids, mouse model

## Abstract

**Background:**

Pancreatic cancer is a highly aggressive malignancy with limited therapeutic options due to rapid tumor progression and poor prognosis. Fecal Microbiota Transplantation (FMT) has emerged as a promising approach to modulate gut microbiota, potentially enhancing the efficacy of conventional treatments.

**Objectives:**

This study evaluates the combined effects of FMT and 5-fluorouracil (5FU) on gut microbiota composition, pancreatic tumor growth, and systemic immune responses in a murine model.

**Methods:**

One hundred female C57BL/6 mice aged 6–8 weeks were randomly divided into five groups (*n* = 20 each): Sham, Model, FMT, 5FU, and FMT + 5FU. Pancreatic tumors were induced via orthotopic implantation of Pan02 cells. FMT was administered orally (0.2 g fecal material) three times per week, starting 2 weeks before tumor implantation. 5FU was administered intraperitoneally at 25 mg/kg body weight twice weekly, beginning one-week post-tumor implantation. Gut microbiota was analyzed via 16S rRNA gene sequencing of fecal samples after 10-week cell implantation. Tumor volumes were measured, and serum cytokine levels were assessed. Short-chain fatty acids (SCFAs) in blood and feces using gas chromatography–mass spectrometry (GC–MS).

**Results:**

The FMT + 5FU group exhibited the smallest average tumor volume, significantly smaller than the Model (*p* < 0.0001) and 5FU groups (*p* = 0.005). FMT alone reduced tumor volume compared to the Model group (*p* < 0.0001). Gut microbiota analysis revealed increased *α* diversity in the FMT group compared to the Model group (*p* < 0.0001). The FMT + 5FU group showed a significant reduction in cytokine levels, including TNF-*α* (*p* = 0.0001) and IL-6 (*p* = 0.012) and increased IL-10 level (*p* < 0.001), compared to the Model group. Plasma and fecal SCFA concentrations were significantly higher in both FMT and FMT + 5FU groups relative to the Model group (*p* < 0.001). Additionally, the FMT + 5FU group had the highest survival rate (50%) after 10-week cell implantation, compared to the Model group (15%).

**Conclusion:**

FMT significantly enhances the efficacy of 5FU in reducing pancreatic tumor growth through gut microbiota modulation.

## Introduction

The trillions of microbes inhabiting the gut regulate digestion, immunity and systemic metabolism—and their imbalance, or dysbiosis, contributes to obesity (Longo et al., 2023; Van Hul and Cani, 2023), diabetes (Bajinka et al., 2023; Crudele et al., 2023), cardiovascular diseases (Nesci et al., 2023), and various forms of cancer (Long et al., 2023; Wong and Yu, 2023). Experimental and clinical data link dysbiosis to disease progression and therapy resistance, suggesting that restoring microbial balance could bolster treatment efficacy (Chu et al., 2023; Hamjane et al., 2023).

Pancreatic ductal adenocarcinoma remains one of the deadliest malignancies, with a five-year survival under 10% despite surgery, targeted agents and chemotherapy (Halbrook et al., 2023; Strickler et al., 2023). 5-Fluorouracil (5FU) is a cornerstone drug (Kawakami et al., 2023), but its benefit is limited by toxicity and emerging resistance. Mounting evidence shows that gut microbes modulate both the efficacy and side-effect profile of chemotherapeutics (Chrysostomou et al., 2023; Lo et al., 2023), opening the door to microbiota-focused adjuvant strategies.

Specific genera—including Akkermansia, Bacteroides, Clostridium, Escherichia, Lactobacillus and Bifidobacterium—have been implicated in pancreatic tumor biology via effects on inflammation, bile-acid metabolism and genotoxin production (Abe et al., 2024; Bai et al., 2023; Kartal et al., 2022; Luo et al., 2020; Pahle et al., 2021; Saeed et al., 2024; Shim et al., 2021; Zhao et al., 2023). Their metabolites, notably short-chain fatty acids (acetate, propionate, butyrate), regulate gut barrier integrity, immune signaling and cancer cell proliferation (Murthy et al., 2024; Panebianco et al., 2022; Perazzoli et al., 2023; Zhong et al., 2024).

Fecal microbiota transplantation (FMT)—the transfer of stool from healthy donors—has proven effective against refractory Clostridioides difficile and shows promise in modulating chemotherapy response (Routy et al., 2023; Xu et al., 2023). Preclinical data suggest FMT can reshape microbial communities to enhance drug sensitivity and reduce toxicity (Bangolo et al., 2023). Here, using an orthotopic Pan02 mouse model, we test whether FMT augments 5FU’s anti-tumor effects by increasing microbial diversity and beneficial taxa, thereby reducing tumor growth and improving survival (Bangolo et al., 2023; de Castilhos et al., 2024).

Our research addresses a critical gap how gut microbiota modulation can influence cancer treatment outcomes. By exploring the interaction between FMT and chemotherapy in a well-established rodent model, this study aims to explore the potential of microbiota-targeted therapies as adjuncts to conventional cancer treatments.

## Methods

### Animals and experimental design

The animal experiments were approved by the Animal Ethics Committee of Shengjing Hospital of China Medical University, Shenyang, China. All procedures were conducted in accordance with the ethical guidelines for the care and use of laboratory animals and were designed to minimize animal suffering. A total of 100 female C57BL/6 mice, aged 6–8 weeks and weighing approximately 20 grams each, were used per group. Initially, the study included 20 mice per group, providing a robust sample size for comparisons across the five groups. However, by the end of the study, the model group was reduced to only 3 surviving mice in the model group, necessitating the use of an equally reduced sample size of 3 mice per group for balanced comparisons. The animals were housed under controlled conditions with a 12-h light/dark cycle and were provided with food and water ad libitum. Before the commencement of the experiment, all animals were allowed to acclimate to their environment for 1 week. The study was divided into five groups, each receiving different treatments.

### FMT

The antibiotic mixture consisted of ampicillin (1 g/L), vancomycin (0.5 g/L), neomycin (1 g/L), and metronidazole (1 g/L), dissolved in drinking water and administered ad libitum to all the mice for 7 days—to uniformly deplete the native gut flora. Antibiotics were withdrawn for 1 week to allow clearance of residual drugs before any downstream intervention. This broad-spectrum cocktail was selected to deplete the endogenous gut microbiota, creating a receptive environment for subsequent FMT engraftment. FMT was initiated 7 days after completing this antibiotic treatment to allow sufficient time for microbial depletion while minimizing residual antibiotic effects that could interfere with the donor microbiota’s colonization. Except for the Sham, Model, and 5FU groups, all other groups received FMT starting 2 weeks before the orthotopic tumor implantation. FMT was administered orally using a gavage method, with each mouse receiving 0.2 grams of fecal material three times per week until the end of the experiment. The fecal samples used for transplantation were obtained from healthy, matched donor mice. These samples were aliquoted into 2 mL centrifuge tubes according to the required number of doses.

All donor mice are housed in the same experimental rooms under standardized conditions (22–24°C, humidity 40–60%, 12-h light/12-h dark cycle (lights on at 06:00, off at 18:00) with minimal-intensity lighting (~130–325 lux), and 10–15 air changes per hour (ACH) with HEPA-filtered airflow). Animals from different cages are not mixed during the study. Fecal samples are collected and processed per cage to avoid cross-contamination and maintain microbiota integrity. Based on past experience, the microbial composition of fecal samples from the same mouse may exhibit slight variations within a one-week timeframe. To mitigate this variability, fresh fecal samples from donor mice are pooled (within cages), homogenized, and processed into a single identical microbial inoculum. This inoculum is aliquoted and stored at –80°C immediately after preparation. Frozen aliquots are thawed once for administration to ensure consistency across experiments, eliminating batch-to-batch variability and temporal confounding factors.

### Orthotopic tumor formation and monitoring

The Pan02 mouse pancreatic cancer cell line, known for its high tumor-forming efficiency, was cultured and used as the tumor source for orthotopic implantation. Mice were anesthetized using a suitable anesthetic protocol, such as a combination of xylazine and ketamine or ether, supplemented with subcutaneous injections of analgesics like carprofen or meloxicam to minimize pain during surgery. For orthotopic tumor implantation, a transverse incision was made on the abdominal wall of each C57BL/6 mouse. Approximately 5 × 10^5^ Pan02 cells were suspended in a mixture of 1/4 volume Matrigel and an equal volume of physiological saline. A volume of 40–60 μL containing around 2 × 10^5^ cells was then injected directly into the pancreas. Tumor formation was typically observable 2–4 weeks post-implantation, with tumors being monitored weekly.

### 5-Fluorouracil treatment

In the 5FU and FMT + 5FU groups, 5-fluorouracil (5FU) was administered as a chemotherapeutic treatment to assess its impact on tumor growth and survival. The 5FU was administered intraperitoneally at a concentration of 25 mg/kg body weight. The treatment was initiated 1 week after the tumor cell transplantation and was continued twice weekly for a period of 10 weeks. This dosing regimen was designed to mimic clinical chemotherapy schedules and to evaluate the long-term effects of 5FU on tumor progression in the presence and absence of fecal microbiota transplantation.

### Pancreatic cancer mouse model and treatment groups

In this study, a pancreatic cancer mouse model was established using tumor cell transplantation. Specifically, mice in the Model group received a surgical procedure during which tumor cells were directly transplanted onto the pancreas, facilitating the development of pancreatic tumors. The Sham group underwent the same surgical procedure without the transplantation of tumor cells, serving as a surgical control to isolate the effects of the surgery from tumor growth. The experimental groups were divided into five distinct cohorts: Sham, Model, FMT, 5FU, and FMT + 5FU. Mice in the FMT group received fecal microbiota transplantation (FMT) post-surgery to assess the influence of gut microbiota on tumor progression. The 5FU group was treated with 5-fluorouracil (5FU), a chemotherapeutic agent, following tumor cell transplantation to evaluate its effectiveness in inhibiting tumor growth. The FMT + 5FU group received a combination of FMT and 5FU treatment to explore the potential synergistic effects of microbiota modulation and chemotherapy on pancreatic cancer.

### Microbiome analysis

To minimize diurnal variation and environmental contamination, fecal samples were collected from each mouse in the five experimental groups (Sham, Model, FMT, 5FU, and FMT + 5FU) at the same time each day (8:00 AM) during the tenth week of treatment. All samples were collected under sterile conditions using autoclaved collection tools and immediately transferred to cryovials, which were subsequently snap-frozen in liquid nitrogen. The samples were then stored at –80°C until DNA extraction, ensuring the preservation of microbial DNA integrity. Alongside the experimental samples, mock community standards (ZymoBIOMICS Microbial Community Standard, Zymo Research, Cat D6300) and blank extraction controls were included to monitor potential contamination during the DNA extraction and sequencing processes.

Genomic DNA was extracted using the QIAamp Fast DNA Stool Mini Kit (Qiagen, Cat 51,604) with an additional bead-beating step for 2 min at 6,000 rpm (using the MP Biomedicals FastPrep-24) to ensure efficient lysis of all bacterial cell types, including Gram-positive bacteria. DNA extraction efficiency was further assessed by comparing the yield and purity of DNA from the experimental samples to that from the mock community standards. DNA concentration and purity were measured using a NanoDrop ND-1000 spectrophotometer (Thermo Fisher Scientific) and fluorometric quantification with the Qubit dsDNA HS Assay Kit (Thermo Fisher Scientific, Cat Q32854). The integrity of the DNA was confirmed via agarose gel electrophoresis on a 1% agarose gel.

The bacterial 16S rRNA gene, specifically the V3–V4 hypervariable regions, was amplified using universal primers (Forward: 5’-CCTACGGGNGGCWGCAG-3′, Reverse: 5’-GACTACHVGGGTATCTAATCC-3′) to target approximately 469 bp of the 16S gene. Each 25 μL PCR reaction contained 12.5 μL of 2X KAPA HiFi HotStart ReadyMix (Roche, Cat KK2601), 0.2 μM of each primer, and 20 ng of template DNA. The PCR conditions were set as follows: initial denaturation at 95°C for 3 min, followed by 35 cycles of 95°C for 30 s, 55°C for 30 s, and 72°C for 45 s, with a final extension at 72°C for 7 min. Each sample was amplified in triplicate to reduce PCR bias, and the pooled PCR products were purified using AMPure XP beads (Beckman Coulter, Cat A63880). Sequencing libraries were prepared with the Nextera XT DNA Library Preparation Kit (Illumina, Cat FC-131-1024) and sequenced on an Illumina MiSeq platform, generating paired-end reads of 2 × 300 bp.

Raw sequencing data were processed using QIIME2 (version 2023.2), where quality control was implemented via the DADA2 plugin, which performs demultiplexing, quality filtering, chimera detection, and denoising. Reads with a Phred quality score below 30 were discarded, and sequences were truncated to 250 bp to ensure high-quality base calls throughout the dataset. Taxonomic assignment was carried out using a pre-trained Naive Bayes classifier based on the SILVA 138 99% OTUs database, with an additional decontamination step using the Decontam package to identify and remove any contaminant sequences. *Α* diversity metrics, including Chao1, Shannon Index, and Faith’s PD, were calculated to evaluate the richness and phylogenetic diversity within samples. *Β* diversity was assessed using weighted UniFrac distances, and ordination was performed using Principal Coordinates Analysis (PCoA) with PERMANOVA employed to test for significant differences between groups.

Functional profiling was conducted using PICRUSt2 (Phylogenetic Investigation of Communities by Reconstruction of Unobserved States), which predicts the metagenome based on the 16S rRNA gene data. The predicted metagenomes were then analyzed to identify pathways and functions that were significantly enriched across the different treatment groups. Statistical analyses of microbiota composition and functional profiles were performed using ANOVA, followed by *post hoc* Tukey’s HSD test, with a significance threshold set at *p* < 0.05. False Discovery Rate (FDR) corrections were applied to control for multiple comparisons. Differential abundance analysis was further complemented by LEfSe (Linear discriminant analysis Effect Size) to identify key bacterial taxa associated with each experimental condition. Visualizations, including heatmaps, bar plots, and microbial co-occurrence networks, were generated using the ggplot2 and pheatmap packages in R (version 4.3.1).

### Tumor volume measurement and body weight monitoring

Tumor volume was measured at the conclusion of the 10-week study period to assess the extent of tumor growth across the different experimental groups. Due to high mortality in the Model group, only 3 mice survived until the 10-week endpoint following cell injection. To ensure balanced group sizes for comparative analyses, 3 mice were randomly selected from each of the other groups, resulting in a final sample size of *n* = 3 per group for tumor volume measurements. Tumor tissues were harvested from these selected mice, and their volumes were calculated using the ellipsoid formula: $V=\frac{4}{3}\pi \times \frac{l}{2}\times \frac{w}{2}\times \frac{h}{2}$ , where l, w, and h represent the length, width, and height of the tumor, respectively, measured with rulers (10ths of a mm). All measurements were performed in triplicate by two independent researchers blinded to the group assignments, and the average values were used for analysis to minimize measurement bias. Tumor volume data are presented as mean ± standard deviation (SD) for each group, with the caveat that the limited sample size may affect the statistical power to detect differences.

Body weight was monitored weekly throughout the 10-week study period to evaluate the impact of tumor growth and the various treatments on the overall health and metabolism of the mice. Body weight measurements were recorded for all mice at baseline (week 0) and every 7 days thereafter, using a calibrated digital scale with a precision of 0.1 g. To account for the reduced sample size at the endpoint, body weight analyses were conducted in two stages: (1) weekly body weight progression was analyzed for all surviving mice in each group over the 10-week period to assess temporal trends in body mass, and (2) final body weights were recorded at the end of the study (week 10) for the randomly selected subset of *n* = 3 mice per group to evaluate cumulative weight changes and treatment effects. The selection of the subset of mice for final body weight analysis was performed randomly using a computer-generated randomization algorithm to avoid selection bias. Body weight data are expressed as mean ± SD, and the weight progression of each group was analyzed to determine the temporal effects of tumor burden and treatments on body mass.

### Survival analysis

Survival rates were assessed over the 10-week period using Kaplan–Meier survival analysis. Mice were monitored daily for signs of morbidity and mortality, and the survival data were recorded accordingly. The Kaplan–Meier curves were generated for each group to visualize the impact of tumor growth and treatment on survival. For the purposes of Kaplan–Meier curve construction, the time-to-event data were grouped into 7-day increments (week 1: days 1–7, week 2: days 8–14, etc.). Comparisons between groups were made to determine the effectiveness of the treatments in extending survival, with particular focus on the potential benefits of FMT, 5FU, and their combination (FMT + 5FU) in improving outcomes in the pancreatic cancer model. The statistical significance of differences in survival between groups was analyzed to validate the results.

To evaluate the diagnostic potential of specific gut microbiota species in distinguishing between different experimental groups, receiver operating characteristic (ROC) curve analysis was conducted. Bacterial taxa were identified through 16S rRNA gene sequencing of fecal samples collected from the mice in the various treatment groups (Sham, Model, FMT, 5FU, and FMT + 5FU). Sequencing reads were processed and assigned to operational taxonomic units (OTUs) using the QIIME2 pipeline. The relative abundance of key bacterial species—Akkermansia, Clostridium, Escherichia, Bacteroides, Lactobacillus, and Bifidobacterium—was calculated for each sample. ROC curves were generated using the pROC package in R, with sensitivity plotted against 1-specificity to assess the species’ ability to correctly classify samples according to their treatment group. The area under the curve (AUC) was calculated for each species to quantify its diagnostic accuracy, with higher AUC values indicating better discriminatory power.

To identify bacterial taxa that were differentially abundant between the experimental groups, a LEfSe (Linear Discriminant Analysis Effect Size) analysis was performed. The relative abundance data from 16S rRNA gene sequencing were processed through the LEfSe algorithm to compute Linear Discriminant Analysis (LDA) scores. These scores were used to rank bacterial genera based on their differential abundance across the groups, with positive LDA scores indicating genera more prevalent in the Model group and associated with disease progression, and negative LDA scores indicating genera more abundant in the treatment groups, associated with protective effects. The analysis was carried out using the LEfSe tool available in the Galaxy platform, with default parameters including an *α* value for the factorial Kruskal-Wallis test among classes set at 0.05 and a logarithmic LDA score threshold set at 2.0 for discriminative features. The results were visualized as a bar chart, highlighting the taxa with the most significant differences in abundance between the groups.

### ELISA analysis

At the end of the 10-week experimental period, serum was extracted from the mice to evaluate circulating levels of inflammatory cytokines, following ethical euthanasia guidelines. Blood was collected via cardiac puncture, allowed to clot at room temperature for 30 min, and then centrifuged at 2,000 g for 10 min at 4°C to isolate the serum, which was carefully harvested (yielding approximately 100–200 μL per mouse) and stored at –80°C until analysis. ELISA kits from Sangon Biotech (Shanghai) Co., Ltd., China, were utilized to quantify the expression of key inflammatory cytokines in the serum, including TNF-α (Cat. No. D721217), IL-1β (Cat. No. D721017), IL-6 (Cat. No. D721022), and IL-10 (Cat. No. D721023).

### Western blot analysis

Western blotting was conducted to assess the expression levels of key inflammatory markers and signaling proteins in the tumor tissues. Tumor tissues were lysed using RIPA buffer (Thermo Fisher Scientific, Cat 89,901) supplemented with protease and phosphatase inhibitors (Sigma-Aldrich, Cat P8340). Protein concentrations were quantified using a bicinchoninic acid (BCA) assay kit (Thermo Fisher Scientific, Cat 23,225). Equal amounts of protein (30–50 μg) were loaded onto 10% SDS-PAGE gels and subjected to electrophoresis. After separation, proteins were transferred onto polyvinylidene difluoride (PVDF) membranes (Millipore, Cat IPVH00010). The membranes were blocked with 5% non-fat milk in Tris-buffered saline with 0.1% Tween 20 (TBST) for 1 h at room temperature to prevent non-specific binding. The membranes were then incubated overnight at 4°C with primary antibodies specific to the proteins of interest, diluted 1:1000 in TBST with 1% non-fat milk. The primary antibodies used were as follows: TNF-*α* (rabbit polyclonal, Abcam, Cat ab6671), IL-1*β* (rabbit polyclonal, Abcam, Cat ab9722), IL-6 (rabbit monoclonal, Cell Signaling Technology, Cat 12,912), and IL-10 (rabbit monoclonal, Cell Signaling Technology, Cat 20,850).

After the overnight incubation with primary antibodies, the membranes were washed three times with TBST and then incubated with horseradish peroxidase (HRP)-conjugated secondary antibodies (anti-rabbit IgG, HRP-linked, Cell Signaling Technology, Cat 7074S) at a dilution of 1:2000 in TBST for 1 h at room temperature. Following the secondary antibody incubation, the membranes were washed again with TBST, and the protein bands were visualized using an enhanced chemiluminescence (ECL) detection system (Thermo Fisher Scientific, Cat 32,106). The intensity of the protein bands was quantified using ImageJ software (NIH, Bethesda, MD). The band intensities were normalized to the corresponding loading control, either β-actin (mouse monoclonal, Sigma-Aldrich, Cat A5441) or GAPDH (rabbit polyclonal, Abcam, Cat ab9485), to correct for variations in protein loading. The relative expression levels were calculated and compared across different experimental groups to elucidate the effects of the treatments on protein expression related to inflammation and signaling pathways in pancreatic cancer.

### Measurement of SCFAs in serum and feces

Evaluating SCFAs in serum and feces provides critical insights into the interplay between gut microbiota, inflammation, and tumor suppression in pancreatic cancer mouse models, and involves systematic sample collection, preparation, extraction, and analysis using gas chromatography–mass spectrometry (GC–MS). For fecal sample collection, fresh fecal pellets should be obtained from each mouse at the end of the 10-week experimental period. Approximately 50–100 mg of fecal pellets per sample should be collected into sterile, pre-weighed microcentrifuge tubes and immediately frozen at –80°C to preserve SCFA integrity. For serum samples, blood should be collected via cardiac puncture or tail vein bleeding under terminal anesthesia at the 10-week endpoint, allowed to clot at room temperature for 30 min, and then centrifuged at 2,000 × g for 10 min at 4°C to separate serum. Approximately 100–200 μL of serum per sample should be transferred into sterile microcentrifuge tubes and stored at –80°C until analysis, ensuring the stability of SCFAs for subsequent processing.

Sample preparation and SCFA extraction differ slightly between fecal and serum samples but follow a standardized protocol to isolate SCFAs such as acetate, propionate, and butyrate for analysis. For fecal samples, thaw the pellets on ice, weigh them, and homogenize in 500 μL of ice-cold deionized water or 0.15 M phosphoric acid (to stabilize SCFAs) using a vortex mixer or bead beater. Centrifuge the homogenate at 13,000 × *g* for 10 min at 4°C to pellet debris, then transfer the supernatant to a new tube and add an internal standard (4-methylvaleric acid at 1 mM) to account for extraction variability. Acidify the supernatant with 100 μL of 50% sulfuric acid to protonate SCFAs, followed by extraction with 1 mL of diethyl ether or ethyl acetate, vortexing vigorously for 1 min, and centrifuging at 3,000 × *g* for 5 min to separate phases. The organic phase, containing SCFAs, should be transferred to a new tube, evaporated under a nitrogen stream to concentrate the sample, and resuspended in 100 μL of ethyl acetate for GC–MS analysis. This meticulous preparation ensures accurate detection of SCFAs in fecal samples.

For serum samples, the extraction process is adapted to the liquid matrix while maintaining precision in SCFA recovery. Thaw serum samples on ice, aliquot 100 μL into a microcentrifuge tube, and add 10 μL of the internal standard (4-methylvaleric acid at 1 mM) to normalize extraction efficiency. Acidify the serum with 20 μL of 50% sulfuric acid to protonate SCFAs, then extract them by adding 200 μL of diethyl ether or ethyl acetate, vortexing vigorously for 1 min, and centrifuging at 3,000 × *g* for 5 min to separate phases. Transfer the organic phase to a new tube, evaporate it under a nitrogen stream to concentrate the SCFAs, and resuspend the residue in 50 μL of ethyl acetate for GC–MS analysis. These steps ensure that SCFAs in serum, which are present at lower concentrations than in feces, are effectively isolated and concentrated for reliable quantification, providing insights into systemic SCFA levels influenced by gut microbiota activity.

GC–MS analysis requires precise instrumentation and calibration to quantify SCFAs accurately across both sample types. Use a gas chromatograph coupled with a mass spectrometer (Agilent 7890B GC with 5977A MSD) equipped with a capillary column suitable for SCFA analysis (Agilent DB-FFAP or HP-INNOWax, 30 m × 0.25 mm × 0.25 μm), with helium as the carrier gas at a flow rate of 1 mL/min and a splitless injection mode with a 1 μL injection volume. The temperature program should start at 50°C (held for 1 min), increase at 10°C/min to 180°C, then at 20°C/min to 240°C (held for 5 min) to elute all compounds, with injector and detector temperatures set to 250°C. Operate the mass spectrometer in electron ionization (EI) mode at 70 eV, using selected ion monitoring (SIM) mode to detect specific ions (m/z 60 for acetic acid, m/z 74 for propionic acid, m/z 88 for butyric acid, m/z 87 for valeric acid, and m/z 101 for 4-methylvaleric acid). Calibration involves preparing a standard mixture of SCFAs (acetic, propionic, butyric, isobutyric, valeric, and isovaleric acids) at concentrations from 0.1 to 10 mM in ethyl acetate, including the internal standard, to establish retention times and generate calibration curves by plotting peak area ratios (SCFA/internal standard) against concentrations. Quantify SCFAs in samples by interpolating peak area ratios from these curves, normalizing to fecal weight (μmol/g) or serum volume (μmol/L).

## Results

### Pancreatic tumor development and progression in a mouse model

**Figure 1:**
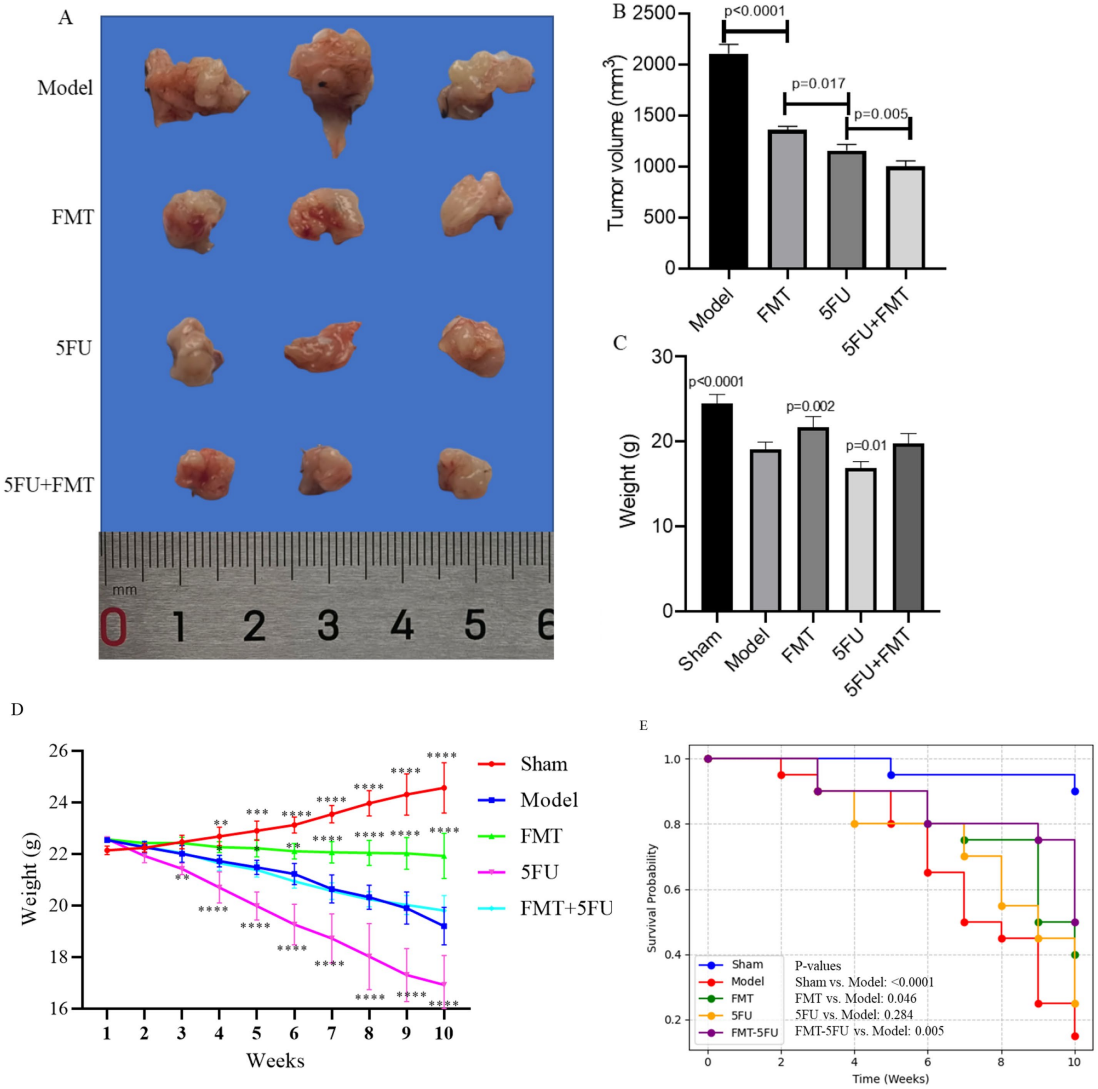
Overview of pancreatic tumor development and progression in a mouse model. **(A)** Representative images of pancreatic tumors isolated from mice after 10 weeks of model establishment, showing varying tumor sizes. **(B)** Quantification of tumor volume across different experimental groups, highlighting significant differences in tumor growth. **(C)** Final body weight of mice after the 10-week period, comparing weight changes among the groups. **(D)** Body weight progression from week 0 to week 10, illustrating weight changes over time in response to the different treatments or conditions. *p < 0.05, *p < 0.01, *p < 0.001 and *p < 0.0001 vs. the model group. **(E)** Kaplan–Meier survival curves representing survival rates of mice over the 10-week period, indicating differences in survival among the groups. The Sham group underwent pancreatic surgery without the transplantation of tumor cells, serving as a surgical control to evaluate the effects of the procedure itself. The Model group had tumor cells transplanted onto the pancreas, establishing a baseline for tumor development without any additional therapeutic intervention. The FMT group received fecal microbiota transplantation (FMT) following the tumor cell transplant, aiming to assess the influence of gut microbiota modulation on tumor growth. The 5FU group was treated with 5-fluorouracil (5FU), a chemotherapy agent, after tumor cell transplantation, to evaluate its effectiveness in inhibiting tumor progression. Finally, the FMT + 5FU group combined fecal microbiota transplantation with 5-fluorouracil treatment to explore the potential synergistic effects of microbiota modulation and chemotherapy in controlling pancreatic cancer. *n* = 3 for each group. There were significant differences if *p* < 0.5.

Figure 1A displays representative pancreatic tumors from different experimental groups after 10 weeks of model establishment, while Figure 1B presents the corresponding tumor volume data (mm^3^). The Model group exhibited the largest tumors, with an average volume of 2109.67 ± 94.05 mm^3^, confirming the aggressive tumor growth in the absence of treatment and the successful establishment of the pancreatic cancer model. In contrast, the Sham group, subjected to pancreatic surgery without tumor cell transplantation, showed no tumor formation, verifying that the surgical procedure alone does not induce tumor growth. The FMT, 5FU, and FMT + 5FU groups displayed visibly smaller tumors, with average volumes of 1361.67 ± 34.93 mm^3^, 1160.33 ± 59.60 mm^3^, and 1006.00 ± 53.62 mm^3^, respectively, indicating that fecal microbiota transplantation (FMT), 5-fluorouracil (5FU) treatment, or their combination effectively reduces tumor size. Statistical analysis revealed significant differences in tumor volume: FMT significantly reduced tumor volume compared to the Model group (*p* < 0.0001), highlighting the potential role of gut microbiota in tumor suppression; 5FU further decreased tumor volume compared to FMT (*p* = 0.017), underscoring its efficacy as a chemotherapeutic agent; and the FMT + 5FU combination yielded the smallest tumor volumes (*p* = 0.005 compared to 5FU alone), suggesting a synergistic effect that enhances tumor suppression.

Figure 1C presents the final body weights of the mice after 10 weeks. The Sham group maintained the highest average body weight, reflecting the absence of tumor burden and the overall health of these mice. The Model group exhibited a significant reduction in body weight, likely due to the metabolic demands and cachexia associated with tumor growth (*p* < 0.0001). The FMT (*p* = 0.002) and 5FUgroups (*p* = 0.01) had higher or lower final body weights than the Model group, indicating that both treatments might have alleviated or induced some of the negative metabolic effects of the tumor. Although body weights in the FMT + 5FU group remained significantly lower than those in the Sham group, they were comparable to or slightly higher than those in the FMT or 5FU groups alone, indicating a potential synergistic effect in improving overall health.

Figure 1D tracks the body weight progression of the mice over the 10-week period. The Sham group consistently gained weight, reflecting normal growth and health. The Model group, however, showed a decline in weight gain, with some weight loss observed in the later weeks, indicating the impact of the tumor on the mice’s metabolism and overall condition. The FMTgroup exhibited a more stable weight curve, with some weight gain, suggesting a protective effect of the gut microbiota against tumor-induced weight loss. The 5FU group showed an initial weight loss, indicating that while 5FU treatment helped control the tumor, it may have had some negative side effects on weight. The FMT + 5FU group had the most stable and steady weight gain, further supporting the potential benefits of combining FMT with chemotherapy.

Figure 1E illustrates the Kaplan–Meier survival curves for the different groups. The Sham group maintained a 90% survival rate by the end of the 10-week period, as expected given the absence of tumor burden. The Model group showed a steady decline in survival, with survival rates dropping to 15% by the end of the study, reflecting the lethal nature of the untreated tumor (*p* < 0.0001 vs. Sham group). The FMT group had improved survival compared to the Model group, with 40% of the mice surviving after 10 weeks, indicating a protective effect of the gut microbiota (*p* = 0.046 vs. Model group). The 5FU group showed a survival rate of 25%, but the difference was not statistically significant (*p* = 0.284 vs. Model group). The FMT-5FU group had the highest survival rate among the treated groups, with 50% of the mice surviving, suggesting that the combination of FMT and 5FU not only suppressed tumor growth but also significantly extended survival (*p* = 0.005 vs. Model group).

### Serum inflammatory cytokines concentration

**Figure 2:**
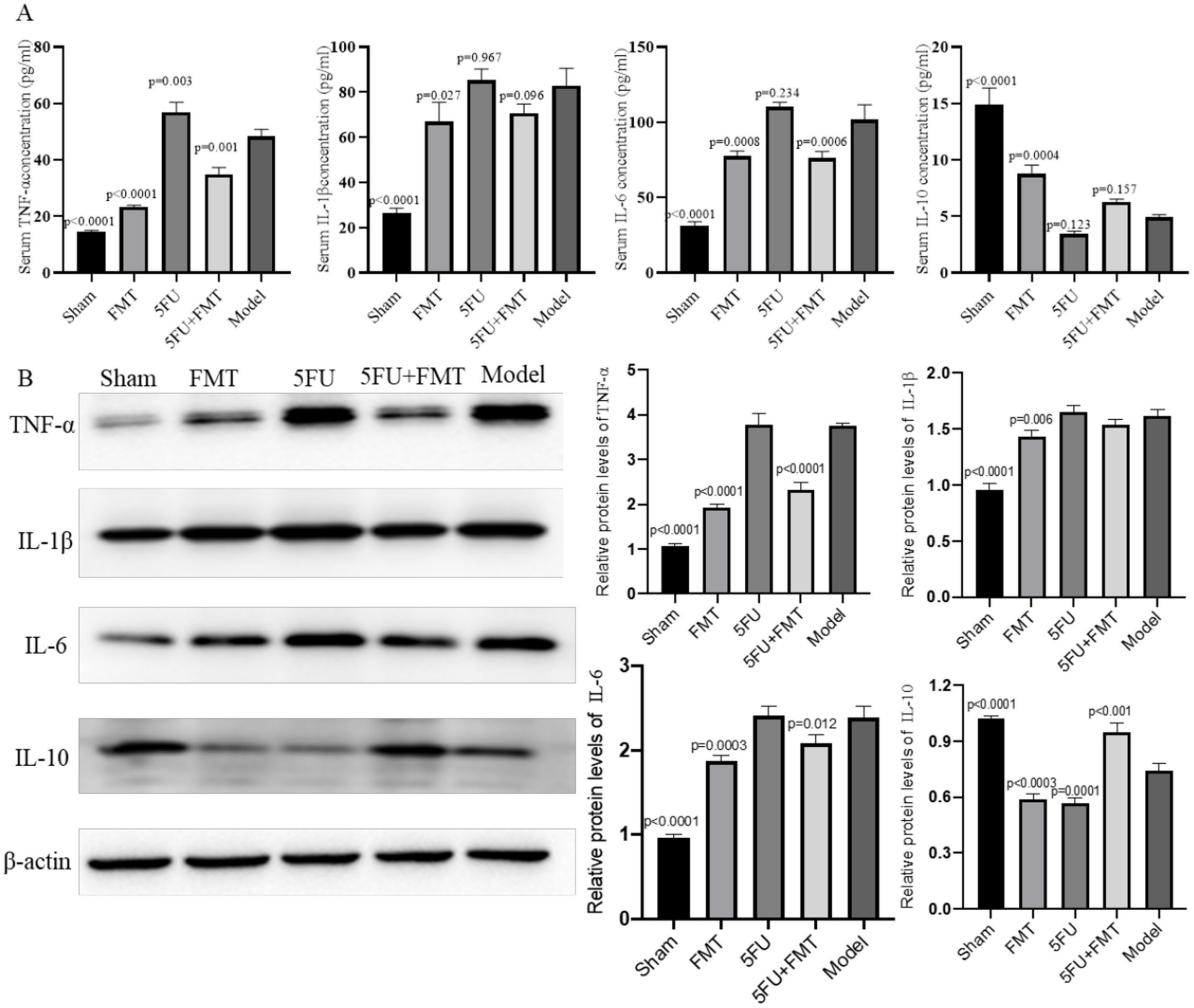
Analysis of inflammatory cytokines and protein expression in serum and tumor tissues. **(A)** Quantitative PCR (qPCR) analysis was performed to measure the levels of inflammatory cytokines TNF-*α*, IL-1β, IL-6, and IL-10 in the serum. The bar graphs represent the relative expression levels of these cytokines across different experimental groups, indicating the impact of various treatments on systemic inflammation. **(B)** Western blot analysis of protein expression levels in tumor tissues, with representative blots shown for key inflammatory markers and signaling molecules. The corresponding bar graphs quantify the relative expression levels, providing insights into the molecular effects of the treatments on tumor biology. *n* = 3 for each group. There were significant differences if *p* < 0.5.

ELISA analysis of serum cytokine concentration reveals significant variations in the levels of TNF-*α*, IL-1β, IL-6, and IL-10 among the different experimental groups (Figure 2A). TNF-α, a key pro-inflammatory cytokine, was significantly elevated in the Model group compared to the Sham group, indicating a heightened inflammatory response due to tumor presence (*p* < 0.0001). The FMT group showed a notable reduction in TNF-α levels compared to the Model group *p* < 0.05, suggesting that FMT may have an anti-inflammatory effect (*p* < 0.0001). The 5FU group exhibited increased TNF-α levels relative to the Model group (*p* = 0.003). The combination treatment group (FMT + 5FU) demonstrated the reduced TNF-α levels, indicating a potential synergistic effect in reducing inflammation (*p* = 0.001).

The analysis of IL-1β, another pro-inflammatory cytokine, mirrored the trends observed with TNF-α. The Model group had significantly elevated IL-1β levels compared to the Sham group (*p* < 0.0001), further confirming the inflammatory milieu associated with tumor development. Treatment with FMT alone reduce IL-1β levels compared to the Model group (*p* = 0.027). The 5FU group also showed an insignificant change in IL-1β levels compared to the Model group (*p* = 0.967), with the FMT + 5FU combination treatment yielding no changes in IL-1β levels (*p* = 0.096).

IL-6, a cytokine often associated with chronic inflammation and cancer progression, was significantly elevated in the Model group relative to the Sham group (*p* < 0.0001). The FMT treatment led to an insignificant change in IL-6 levels (*p* = 0.0008), while the 5FU treatment resulted in no significant change either (*p* = 0.234). On the other hand, IL-10, an anti-inflammatory cytokine, was significantly lower in the Model group compared to the Sham group (*p* < 0.0001). FMT treatment slightly increased IL-10 levels (*p* = 0.0004), while 5FU treatment led to an insignificant reduction (*p* = 0.123). The combination treatment (FMT + 5FU) resulted in an insignificant increase (*p* = 0.157).

### Relative protein levels of inflammatory cytokines

The Western blot analysis shows differential expression of key inflammatory markers and signaling proteins across the experimental groups (Figure 2B). The Model group displayed heightened expression of pro-inflammatory proteins, consistent with the elevated cytokine levels observed in the qPCR analysis. Both the FMT and 5FU groups showed reduced expression of these proteins compared to the Model group, with the FMT + 5FU group exhibiting the lowest expression levels, suggesting that the combination treatment most effectively downregulates inflammatory signaling pathways. The bar graphs quantifying the protein expression levels indicate statistically significant reductions in pro-inflammatory protein levels in the FMT, and FMT + 5FU groups compared to the Model group, with the combination treatment group showing the greatest reduction *p* < 0.01. These results further support the hypothesis that combining FMT with chemotherapy not only mitigates inflammation but also impacts key signaling pathways involved in tumor progression.

### FMT improved SCFA profiles

Analysis of SCFA concentrations in fecal samples across the experimental groups provides valuable insights into the gut microbiota’s role in pancreatic cancer progression and treatment response, supporting SCFA evaluation to better understand underlying mechanisms (Table 1). The Sham group exhibited the highest fecal SCFA levels, with acetate at 65.3 ± 4.2 μmol/g (*p* = 0.0002 vs. Model), propionate at 25.8 ± 2.1 μmol/g (*p* = 0.0007), butyrate at 20.4 ± 1.8 μmol/g (*p* = 0.0003), and total SCFAs at 111.5 ± 6.5 μmol/g (*p* = 0.0001), reflecting robust microbial fermentation in a healthy gut environment. In contrast, the Model group, representing untreated pancreatic cancer, showed significantly reduced SCFA concentrations (acetate: 35.7 ± 3.1 μmol/g; propionate: 15.2 ± 1.4 μmol/g; butyrate: 10.9 ± 1.2 μmol/g; total SCFAs: 61.8 ± 4.7 μmol/g), indicative of microbial dysbiosis linked to tumor progression. The FMT group demonstrated a significant restoration of SCFA levels compared to the Model group (acetate: 58.9 ± 3.8 μmol/g, *p* = 0.0008; propionate: 22.6 ± 1.9 μmol/g, *p* = 0.0021; butyrate: 18.2 ± 1.6 μmol/g, *p* = 0.0010; total SCFAs: 99.7 ± 5.9 μmol/g, *p* = 0.0004), suggesting that fecal microbiota transplantation enhances SCFA production, potentially contributing to its anti-tumor effects as observed in tumor volume reductions. The 5FU group displayed the lowest SCFA concentrations (acetate: 28.4 ± 2.5 μmol/g, *p* = 0.0696; propionate: 12.3 ± 1.1 μmol/g, *p* = 0.1012; butyrate: 8.7 ± 0.9 μmol/g, *p* = 0.1216; total SCFAs: 49.4 ± 3.6 μmol/g, *p* = 0.0712), with no significant difference from the Model group, highlighting chemotherapy’s detrimental impact on microbial function. The FMT + 5FU group showed intermediate SCFA levels (acetate: 52.1 ± 3.5 μmol/g, *p* = 0.0034; propionate: 20.1 ± 1.7 μmol/g, *p* = 0.0118; butyrate: 16.8 ± 1.4 μmol/g, *p* = 0.0042; total SCFAs: 89.0 ± 5.2 μmol/g, *p* = 0.0016), indicating that FMT mitigates 5FU’s negative effects on SCFA production.

**Table 1:** Short-chain fatty acid concentrations in feces (μmol/g wet weight).

Group	Acetate (μmol/L)	*p*-value	Propionate (μmol/L)	*p*-value	Butyrate (μmol/L)	*p*-value	Total SCFAs	*p*-value
Sham	65.3 ± 4.2	0.0002	25.8 ± 2.1	0.0007	20.4 ± 1.8	0.0003	111.5 ± 6.5	0.0001
Model	35.7 ± 3.1	–	15.2 ± 1.4	–	10.9 ± 1.2	–	61.8 ± 4.7	–
FMT	58.9 ± 3.8	0.0008	22.6 ± 1.9	0.0021	18.2 ± 1.6	0.0010	99.7 ± 5.9	0.0004
5FU	28.4 ± 2.5	0.0696	12.3 ± 1.1	0.1012	8.7 ± 0.9	0.1216	49.4 ± 3.6	0.0712
FMT + 5FU	52.1 ± 3.5	0.0034	20.1 ± 1.7	0.0118	16.8 ± 1.4	0.0042	89.0 ± 5.2	0.0016

Data are presented as mean ± standard error of the mean (SEM), *n* = 3 per group. *p* < 0.05 vs. the model group indicates statistical significance.

Serum SCFA concentrations complement the fecal data and align with the reviewer’s recommendation to evaluate SCFAs in serum and feces to ensure model accuracy and elucidate systemic mechanisms of tumor modulation (Table 2). The Sham group exhibited the highest serum SCFA levels, with acetate at 450.2 ± 25.3 μmol/L (*p* = 0.0003 vs. Model), propionate at 35.7 ± 2.8 μmol/L (*p* = 0.0018), butyrate at 15.9 ± 1.2 μmol/L (*p* = 0.0007), and total SCFAs at 501.8 ± 27.4 μmol/L (*p* = 0.0002), consistent with efficient systemic transport of SCFAs from a healthy gut microbiota. The Model group showed significantly reduced serum SCFAs (acetate: 250.8 ± 18.6 μmol/L; propionate: 20.4 ± 1.9 μmol/L; butyrate: 8.2 ± 0.9 μmol/L; total SCFAs: 279.4 ± 20.1 μmol/L), reflecting the systemic consequences of microbial dysbiosis in pancreatic cancer. FMT treatment significantly increased serum SCFA concentrations compared to the Model group (acetate: 400.6 ± 22.1 μmol/L, *p* = 0.0012; propionate: 30.9 ± 2.5 μmol/L, *p* = 0.0064; butyrate: 13.8 ± 1.1 μmol/L, *p* = 0.0023; total SCFAs: 445.3 ± 24.6 μmol/L, *p* = 0.0008), suggesting that FMT enhances systemic SCFA availability, potentially reducing inflammation and supporting tumor suppression as observed in survival and cytokine data. The 5FU group exhibited the lowest serum SCFA levels (acetate: 200.3 ± 15.7 μmol/L, *p* = 0.0865; propionate: 15.8 ± 1.6 μmol/L, *p* = 0.0921; butyrate: 6.5 ± 0.7 μmol/L, *p* = 0.1397; total SCFAs: 222.6 ± 17.2 μmol/L, *p* = 0.0793), with no significant difference from the Model group, indicating that 5FU’s adverse effects extend systemically. The FMT + 5FU group showed intermediate serum SCFA concentrations (acetate: 350.9 ± 20.4 μmol/L, *p* = 0.0056; propionate: 28.1 ± 2.3 μmol/L, *p* = 0.0152; butyrate: 12.4 ± 1.0 μmol/L, *p* = 0.0068; total SCFAs: 391.4 ± 22.8 μmol/L, *p* = 0.0031), demonstrating that FMT counteracts 5FU-induced SCFA depletion.

**Table 2:** Short-chain fatty acid concentrations in serum (μmol/L).

Group	Acetate (μmol/L)	*p*-value	Propionate (μmol/L)	*p*-value	Butyrate (μmol/L)	*p*-value	Total SCFAs	*p*-value
Sham	450.2 ± 25.3	0.0003	35.7 ± 2.8	0.0018	15.9 ± 1.2	0.0007	501.8 ± 27.4	0.0002
Model	250.8 ± 18.6	–	20.4 ± 1.9	–	8.2 ± 0.9	–	279.4 ± 20.1	–
FMT	400.6 ± 22.1	0.0012	30.9 ± 2.5	0.0064	13.8 ± 1.1	0.0023	445.3 ± 24.6	0.0008
5FU	200.3 ± 15.7	0.0865	15.8 ± 1.6	0.0921	6.5 ± 0.7	0.1397	222.6 ± 17.2	0.0793
FMT + 5FU	350.9 ± 20.4	0.0056	28.1 ± 2.3	0.0152	12.4 ± 1.0	0.0068	391.4 ± 22.8	0.0031

Data are presented as mean ± standard error of the mean (SEM), *n* = 3 per group. *p* < 0.05 vs. the model group indicates statistical significance.

### Gut microbiota characters among different groups

**Figure 3:**
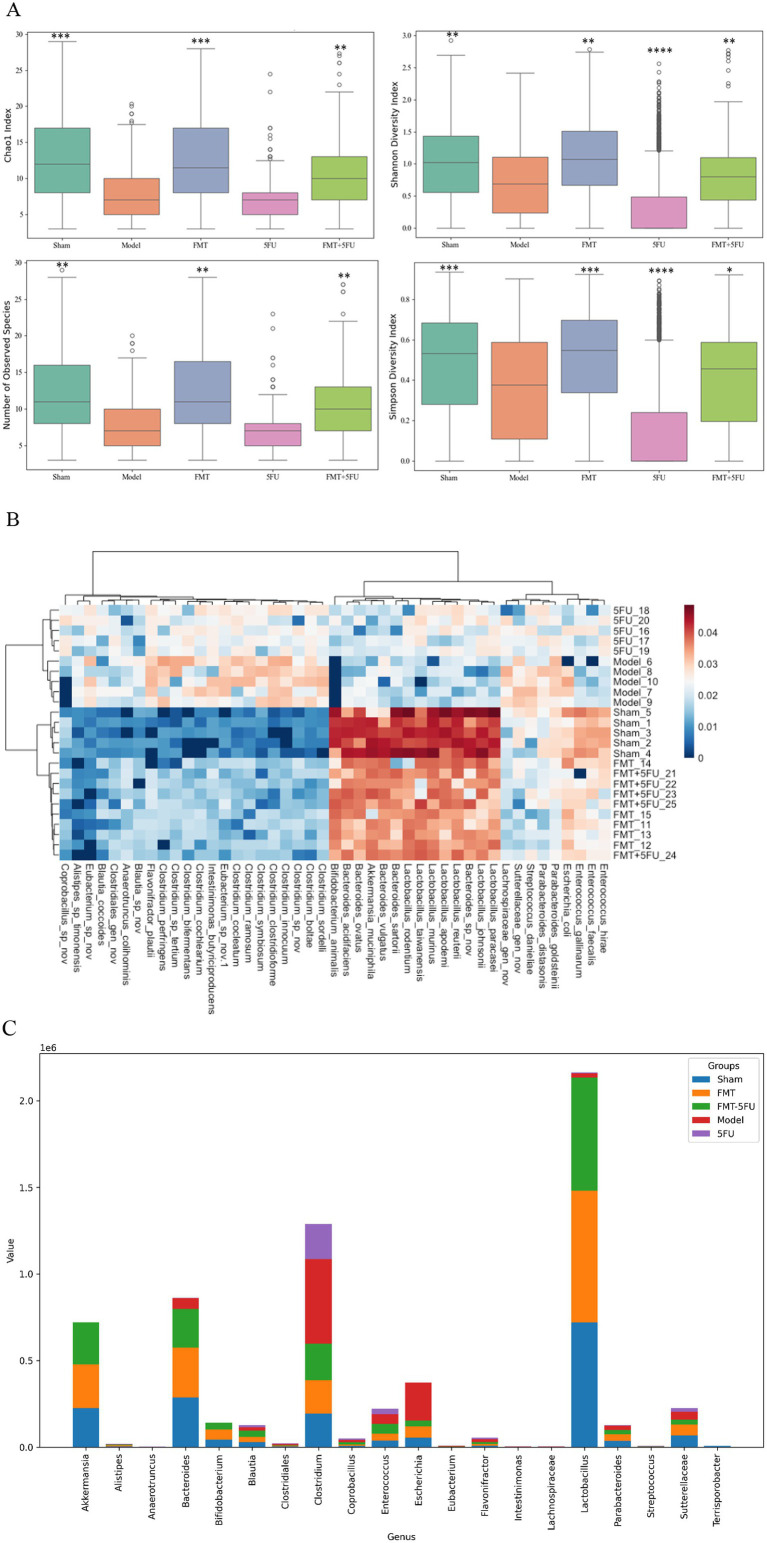
Analysis of gut microbiota diversity and composition across experimental groups. **(A)** Boxplots representing α diversity indices, including Chao1, Observed Species, Shannon Index, and Simpson Index, across the five experimental groups (Sham, Model, FMT, 5FU, and FMT + 5FU). These indices provide insights into the richness and evenness of the gut microbiota, indicating significant differences in microbial diversity among the groups. *p < 0.05, *p < 0.01, *p < 0.001 and *p < 0.0001 vs. the model group. **(B)** Heatmap illustrating the relative abundance of gut microbiota at the genus level across the five groups. The heatmap highlights distinct microbial signatures associated with each treatment, with clustering patterns reflecting the similarities and differences in microbial composition. **(C)** Bar chart showing the proportional distribution of specific gut microbiota genus among the five groups. The chart reveals how different treatments influence the prevalence of various bacterial genera, offering a detailed view of the microbiota shifts in response to the experimental interventions. *n* = 3 for each group. There were significant differences if *p* < 0.5.

Figure 3A presents boxplots of four key *α*-diversity indices—Chao1, Observed Species, Shannon Index, and Simpson Index—across the five experimental groups (Sham, Model, FMT, 5FU, and FMT + 5FU). The Chao1 index, a measure of species richness, was significantly higher in the Sham (*p* < 0.001), FMT (*p* < 0.001), and FMT + 5FU (*p* < 0.01) groups compared to the Model group, with the FMT group exhibiting the highest richness, suggesting that fecal microbiota transplantation markedly enhances microbial diversity. Similarly, the Observed Species count, another richness metric, was significantly elevated in the Sham (*p* < 0.01), FMT (*p* < 0.01), and FMT + 5FU (*p* < 0.01) groups relative to the Model group, reinforcing the impact of tumor burden in reducing microbial richness in the Model group. The Shannon Index, which accounts for both richness and evenness, showed significantly higher diversity in the Sham (*p* < 0.01), FMT (*p* < 0.01), 5FU (*p* < 0.0001), and FMT + 5FU (*p* < 0.01) groups compared to the Model group, indicating more evenly distributed microbial communities in these groups, with the 5FU group showing the most pronounced difference. The Simpson Index, also reflecting richness and evenness, was significantly higher in the Sham (*p* < 0.001), FMT (*p* < 0.001), 5FU (*p* < 0.0001), and FMT + 5FU (*p* < 0.05) groups compared to the Model group, further confirming the adverse effects of chemotherapy on microbial diversity in the 5FU group, albeit with the strongest significance. Notably, the FMT + 5FU group exhibited moderate improvements in diversity across all indices compared to the 5FU group alone, suggesting that FMT may partially mitigate the negative impact of chemotherapy on gut microbiota.

Figure 3B provides a heatmap illustrating the relative abundance of gut microbiota at the genus level across the five experimental groups. The heatmap reveals distinct microbial profiles associated with each group, with clear clustering patterns. The Sham and FMT groups show a higher abundance of beneficial genera such as Lactobacillus and Bifidobacterium, which are known for their health-promoting properties. In contrast, the Model group displays an increased relative abundance of potentially pathogenic genera such as Clostridium and Bacteroides, reflecting the dysbiotic state induced by the tumor. The 5FU group shows a marked reduction in microbial diversity with increased abundance of Enterococcus and Streptococcus, genera often associated with antibiotic use and chemotherapy. The FMT + 5FU group demonstrates a more balanced microbial composition, with some recovery of beneficial genera, indicating that FMT may help restore microbial balance in the context of chemotherapy.

The analysis of microbial genus abundance across different experimental groups (sham3, FMT3, FMT-5FU3, Model3, and 5FU3) revealed distinct compositional profiles, as evidenced by the differential abundance of key genera (Figure 3C). In the sham3 group, Akkermansia (7.22 × 10^5^) and Bacteroides (8.62 × 10^5^) exhibited the highest abundance, followed by Clostridium (1.29 × 10^6^), indicating a dominance of mucin-degrading and butyrate-producing taxa, which are typically associated with a healthy gut microbiome. Conversely, the FMT3 group showed a significant reduction in Akkermansia (2.54 × 10^5^) and Bacteroides (2.88 × 10^5^), with a notable decrease in Clostridium (1.94 × 10^5^), suggesting a potential shift in microbial dynamics post-fecal microbiota transplantation (FMT). The FMT-5FU3 group, which combined FMT with 5-fluorouracil (5FU) treatment, displayed intermediate levels of Akkermansia (2.42 × 10^5^) and Bacteroides (2.23 × 10^5^), with a slight increase in Clostridium (2.11 × 10^5^) compared to FMT3, indicating a partial restoration of microbial diversity. In the Model3 group, representing a disease or dysbiosis model, there was a drastic reduction in Akkermansia (4.74 × 10^2^) and Bacteroides (6.16 × 10^4^), alongside a high abundance of Clostridium (4.88 × 10^5^), reflecting a disrupted microbial ecosystem. The 5FU3 group, treated solely with 5FU, showed the lowest abundance of Akkermansia (1.93 × 10^2^) and Bacteroides (2.09 × 10^3^), with Clostridium (2.03 × 10^5^) remaining dominant, underscoring the profound impact of chemotherapeutic agents on microbial composition. Notably, genera such as Lactobacillus (highest in sham3 at 2.16 × 10^6^) and Escherichia (highest in sham3 at 3.73 × 10^5^) also exhibited significant variations, highlighting the differential effects of FMT, 5FU, and disease states on gut microbial ecology.

### Diagnostic performance and differential abundance of gut microbiota in pancreatic cancer mouse model

**Figure 4:**
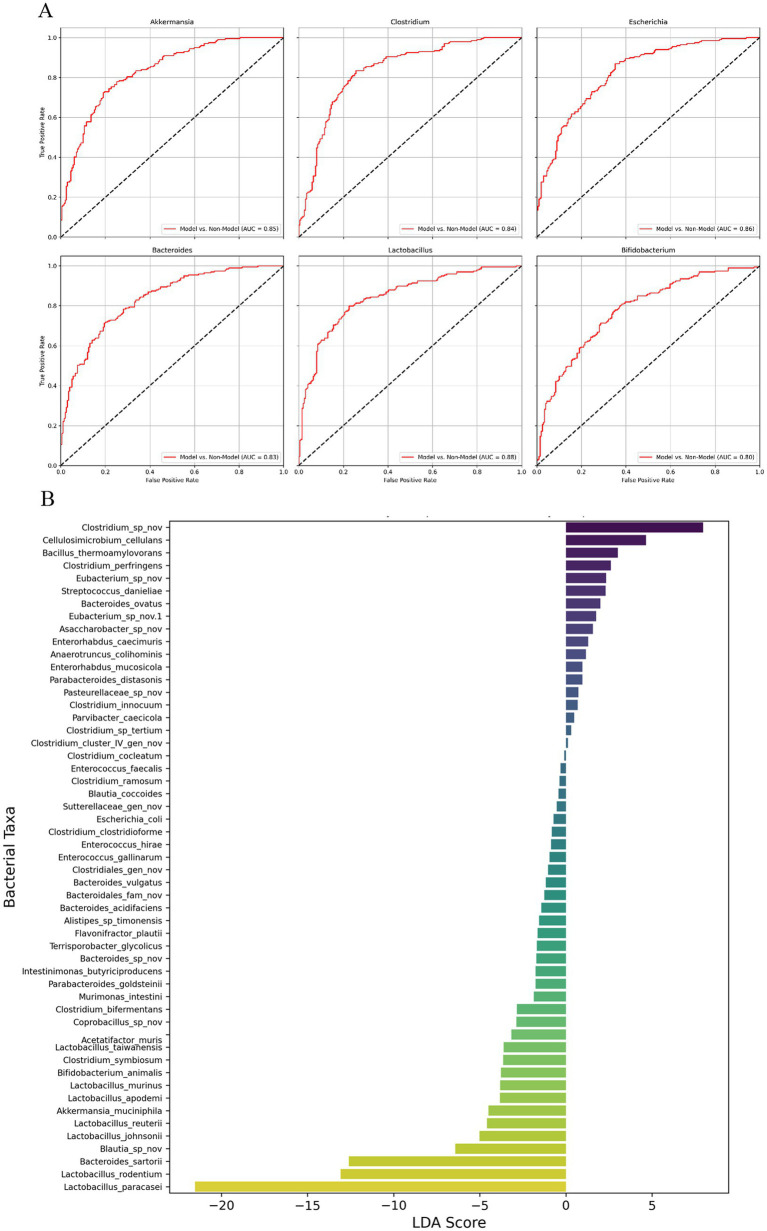
ROC curve analysis and LEfSe-like analysis of gut microbiota in pancreatic cancer mouse model. **(A)** Receiver Operating Characteristic (ROC) curves for six bacterial species (Akkermansia, Clostridium, Escherichia, Bacteroides, Lactobacillus, and Bifidobacterium) in the pancreatic cancer mouse model, showing the diagnostic performance of these species in distinguishing between different treatment groups. **(B)** LEfSe-like analysis displaying the Linear Discriminant Analysis (LDA) scores for various bacterial taxa, highlighting the most differentially abundant genera between the experimental groups. The LDA scores represent the magnitude of the differences, with Clostridium species showing the highest positive LDA score, suggesting a strong association with disease progression, while Lactobacillus and Bifidobacterium are more prevalent in groups with potential protective effects. *n* = 3 for each group.

Figure 4A represents AUC values for six species: Lactobacillus, 0.88; Escherichia, 0.86; Akkermansia, 0.85; Clostridium, 0.84; Bacteroides, 0.83 and Bifidobacterium, 0.80. The results also suggest that these species are also valuable indicators of the gut microbiota’s response to different treatments in the pancreatic cancer model. The high AUC values across these species underscore the importance of gut microbiota composition in reflecting the disease and treatment status in this model.

Figure 4B presents the results of a LEfSe analysis, which identifies the most differentially abundant bacterial taxa between the different experimental groups, represented by Linear Discriminant Analysis (LDA) scores. The LDA scores reflect the magnitude of the difference in abundance for each bacterial genus, with positive scores indicating taxa more abundant in the Model group (associated with disease progression) and negative scores indicating taxa more prevalent in treatment groups (associated with potential protective effects). Clostridium species displayed the highest positive LDA score, reaching over 4.0, highlighting their strong association with tumor progression and their potential role in fostering a pro-tumorigenic environment within the gut microbiota of the Model group. Other genera such as Bacillus thermophilus and Enterococcus also had positive LDA scores around 3.0, indicating their increased presence in the Model group and possible contributions to the dysbiotic state linked to cancer progression. Conversely, genera such as Lactobacillus and Bifidobacterium demonstrated significantly negative LDA scores, with Lactobacillus reaching nearly-5.0 and Bifidobacterium around-4.0. These negative LDA scores indicate that these beneficial genera are more prevalent in treatment groups such as FMT and FMT + 5FU, which are associated with protective effects against tumor growth. The substantial presence of these beneficial bacteria in the treatment groups suggests their involvement in restoring gut microbial balance and potentially contributing to the observed anti-tumor effects.

Overall, the LEfSe-like analysis highlights distinct microbial signatures associated with different disease states and treatments, providing critical insights into the role of gut microbiota in pancreatic cancer progression and response to therapy. The clear delineation between harmful and beneficial bacterial taxa underscores the potential of gut microbiota modulation as a therapeutic strategy in managing pancreatic cancer.

## Discussion

The FMT + 5FU group exhibited the smallest tumor volumes (*p* < 0.0001 vs. Model, *p* = 0.005 vs. 5FU), with FMT alone also reducing tumors (*p* < 0.0001 vs. Model), alongside increased gut microbial *α* diversity in both FMT and FMT + 5FU groups (*p* < 0.0001 vs. Model); FMT + 5FU significantly reduced pro-inflammatory TNF-*α* (*p* = 0.001) with trends toward lower IL-6 and higher IL-10, while both groups showed elevated SCFA levels in feces (FMT: 99.7 ± 5.9 μmol/g, *p* = 0.0004; FMT + 5FU: 89.0 ± 5.2 μmol/g, *p* = 0.0016 vs. Model: 61.8 ± 4.7 μmol/g) and serum (FMT: 445.3 ± 24.6 μmol/L, *p* = 0.0008; FMT + 5FU: 391.4 ± 22.8 μmol/L, *p* = 0.0031 vs. Model: 279.4 ± 20.1 μmol/L), indicating improved microbial function; survival was highest in FMT + 5FU (50%) compared to Model (15%), suggesting FMT enhances 5FU efficacy through microbiota modulation, reducing inflammation and boosting beneficial metabolites like SCFAs.

### FMT regulates gut microbiota

FMT treatment led to increased α diversity in the gut microbiota of both the FMT and FMT + 5FU groups compared to the Model and 5FU groups, indicating a restoration of microbial balance. *Α* diversity is a key indicator of a healthy microbiota, and its increase is often associated with improved health outcomes. The recovery of beneficial bacterial genera, such as *Lactobacillus* and *Bifidobacterium*, in the FMT-treated groups further supports the idea that FMT can counteract chemotherapy-induced dysbiosis. These microbes are known to play protective roles in the host, potentially through mechanisms such as modulation of immune responses, enhancement of mucosal barrier function, and production of anti-inflammatory short-chain fatty acids (Li et al., 2023). These findings align with previous studies demonstrating FMT’s ability to reverse chemotherapy-induced gut dysbiosis, highlighting its potential as a therapeutic tool to restore microbial homeostasis (Yu et al., 2023).

### FMT improves the inflammatory microenvironment

Our results show a marked decrease in pro-inflammatory cytokines, such as TNF-α and IL-6, in the FMT + 5FU group compared to the Model and 5FU groups. This reduction suggests that FMT can create a more anti-inflammatory systemic environment, which is essential for mitigating tumor progression, as chronic inflammation is often associated with cancer advancement (Caronni et al., 2023). The decrease in pro-inflammatory cytokines likely stems from the restoration of beneficial gut microbes that produce anti-inflammatory metabolites, such as short-chain fatty acids, and enhance mucosal barrier function. These findings are consistent with prior research indicating that FMT can reduce inflammatory responses in chemotherapy-treated models, underscoring its potential to create conditions less favorable for tumor growth (Chai et al., 2023).

### FMT combined with 5-FU enhance chemotherapy’s anti-tumor efficacy while reducing its side effects

Our findings demonstrate that the FMT + 5FU group exhibited the most substantial reduction in tumor volume compared to other groups, alongside an increase in gut microbial diversity and improved survival rates. These results suggest that FMT enhances the efficacy of 5FU, potentially by improving drug metabolism and host immune responses through gut microbiota modulation (Gori et al., 2019) (Table 3). The significant reduction in tumor burden in the FMT + 5FU group highlights the potential of gut microbiota modulation as an adjunctive therapy in cancer treatment, aligning with previous studies that have shown similar enhancements in chemotherapy efficacy (Caronni et al., 2023). Furthermore, FMT alleviated 5FU-induced side effects, such as intestinal injury, inflammation, and muscle wasting, as evidenced by our data and supported by prior research (Chang et al., 2020; Chen et al., 2020a; Le Bastard et al., 2018). For instance, FMT has been shown to restore gut microbiota composition, reduce 5FU-induced mucosal damage, and improve nutritional status, thereby mitigating chemotherapy toxicity (Li et al., 2023).

**Table 3:** The interplay between FMT and 5-FU.

Authors & year	Main contribution	Methods	Conclusion
Chang et al. (2020)	Fecal microbiota transplantation prevents intestinal injury, upregulation of toll-like receptors, and 5-fluorouracil/oxaliplatin-induced toxicity in colorectal cancer	FMT administered to mice treated with FOLFOX; monitored intestinal injury, toll-like receptors, goblet cells, and intestinal mucositis after chemotherapy, using histological and molecular analysis.	FMT alleviated FOLFOX-induced intestinal toxicity and restored gut microbiota, without causing bacteremia. Potential mechanism involves gut microbiota TLR-MyD88-NF-kappaB signaling. This suggests FMT could help mitigate 5-FU-induced toxicity in cancer patients.
Chen et al. (2020a)	The gut microbiota attenuates muscle wasting by regulating energy metabolism in chemotherapy-induced malnutrition rats	Chemotherapy-induced 5-Fu rats assessed for muscle wasting, microbiota composition, and metabolic changes; fecal microbiota transplantation (FMT) from healthy rats.	Gut microbiota regulates muscle metabolism and energy production in chemotherapy-induced malnutrition, with FMT from healthy rats improving nutritional status and muscle function, and inhibiting inflammation. Highlights the potential of FMT to combat 5-FU-induced side effects such as muscle wasting.
Chen et al. (2020b)	Berberine regulates fecal metabolites to ameliorate 5-fluorouracil induced intestinal mucositis through modulating gut microbiota	Rats treated with Berberine (BBR) and 5-Fu to assess intestinal mucositis, gut microbiota composition, and fecal metabolites using metabolic profiling.	Berberine improved gut health and metabolism by increasing beneficial metabolites and modifying gut microbiota in 5-Fu-treated rats. FMT from Berberine-treated rats ameliorated intestinal mucosal injury, suggesting Berberine’s potential to influence microbiota composition for therapeutic benefits in 5-FU-related complications.
Chen et al. (2022)	Reactive granulopoiesis depends on T-cell production of IL-17A and neutropenia-associated alteration of gut microbiota	Mouse models of neutropenia and hematopoietic stem cell transplantation; FMT from neutropenic mice to study neutrophil recovery.	Reactive granulopoiesis, induced by neutropenia, was enhanced by gut microbiota, with T-cell IL-17A production being key for neutrophil recovery post chemotherapy. Gut decontamination inhibited this process, supporting a role for the microbiota in immune recovery after 5-FU treatment.
Cheung et al. (2020)	Discovery of an interplay between the gut microbiota and esophageal squamous cell carcinoma in mice	FMT to antibiotic-treated xenograft-bearing mice, followed by chemotherapy with cisplatin and 5-FU, studying liver metastasis and microbiota changes.	Gut microbiota impacts esophageal cancer (EC) metastasis, with FMT influencing anti-metastatic efficacy of chemotherapy and medicinal herbs. Gut microbiota modulation offers potential for improving EC treatment strategies, especially in cases where 5-FU is used.
Gong et al. (2019)	Neohesperidin prevents colorectal tumorigenesis by altering the gut microbiota	Gut microbiota composition assessed in mice treated with Neohesperidin (NHP); FMT used to examine microbiota-mediated tumorigenesis prevention.	Neohesperidin altered gut microbiota composition and inhibited colorectal tumorigenesis, suggesting that changes in microbiota can mediate its chemopreventive effects in conjunction with chemotherapy agents like 5-FU.
Gori et al. (2019)	Gut microbiota and cancer: How gut microbiota modulates activity, efficacy and toxicity of antitumoral therapy	Review of studies on how gut microbiota influences cancer therapy, including chemotherapy, targeted therapy, and immunotherapy.	Gut microbiota plays a significant role in modulating the efficacy and toxicity of various cancer therapies, including 5-FU. Targeting microbiota could enhance treatment outcomes and mitigate side effects.
Le Bastard et al. (2018)	Fecal microbiota transplantation reverses antibiotic and chemotherapy-induced gut dysbiosis in mice	Antibiotic and chemotherapy treatment of mice followed by FMT to restore gut microbiota; sequencing and analysis of microbial diversity and richness.	FMT reversed chemotherapy and antibiotic-induced gut dysbiosis, restoring beneficial gut species and improving intestinal health, suggesting a potential therapeutic strategy for chemotherapy-induced gut dysfunction, particularly in 5-FU-treated patients.
Li et al. (2017)	Alteration of gut microbiota and inflammatory cytokine/chemokine profiles in 5-fluorouracil induced intestinal mucositis	5-Fu-treated mice analyzed for gut microbiota composition, inflammatory cytokines, and chemokines in serum and colon.	5-Fu treatment significantly altered gut microbiota and inflammatory profiles. FMT from healthy mice prevented intestinal damage and weight loss, indicating that microbiota manipulation, such as via FMT, may alleviate 5-Fu-induced mucositis.
Sougiannis et al. (2019)	Impact of 5 fluorouracil chemotherapy on gut inflammation, functional parameters, and gut microbiota	Mice treated with 5-Fu, followed by FMT from 5-Fu treated mice to control group; assessment of gut inflammation and functional parameters.	5-Fu chemotherapy altered gut microbiota composition and induced inflammation, with FMT modulating these effects and influencing functional parameters like grip strength, suggesting that microbiota plays a role in chemotherapy-induced dysfunction and could be targeted with FMT to mitigate 5-FU side effects.

### Implications

The implications of this study are far-reaching, as they suggest that the gut microbiota could be a critical determinant of cancer treatment efficacy. By modulating the gut microbiota through interventions like FMT, it may be possible to enhance the therapeutic effects of existing chemotherapeutic agents, such as 5FU, and reduce their side effects. This approach could pave the way for personalized medicine strategies that consider an individual’s gut microbiota composition when designing cancer treatment regimens. The ability to modulate the gut microbiota also opens new avenues for improving the prognosis of patients with difficult-to-treat cancers, such as pancreatic cancer, where conventional therapies often yield poor outcomes (Chen et al., 2024).

### Limitations and future work

First, the use of a mouse model of pancreatic cancer, while informative, may not fully capture the complexity of human disease, where factors such as diet, genetics, and environmental exposures could influence gut microbiota-chemotherapy interactions. Future research should investigate the effects of FMT in combination with other chemotherapeutic agents and across different cancer models to determine the broader applicability of these benefits (Chrysostomou et al., 2023). Moreover, while our study highlights FMT’s role in enhancing 5FU efficacy through gut microbiota modulation, the specific mechanisms and key bacterial taxa involved remain unclear. Future studies should employ metagenomic and metabolomic analyses to elucidate the functional changes within the gut microbiota that drive these effects.

A significant limitation of this study is the small sample size of only three mice per group, which severely restricts the statistical power and reliability of the findings, particularly for ROC curve analysis of gut microbiota as biomarkers. The small sample size also results in wide confidence intervals, indicating high uncertainty in the estimates, and precludes robust internal validation (cross-validation) or external validation in an independent cohort, limiting the generalizability of the results. Another significant limitation of this study was the high mortality rate observed across the groups, which substantially reduced the sample size and potentially limited the statistical power of the analyses. Although we observed promising synergy between FMT and 5-FU, substantial attrition in the tumor-bearing cohorts reduced our final sample to just three animals per group; consequently, all tumor volume and SCFA measurements were based on *n* = 3. This small, selective subset undermines statistical power and increases the likelihood of both type I and type II errors, potentially exaggerating treatment effects or obscuring more nuanced microbiota–immune interactions. Moreover, the survivors may not be representative of the initial cohort (selection bias), further constraining the robustness and generalizability of our findings. Future experiments employing larger initial group sizes, optimized dosing regimens to reduce early mortality, and independent validation cohorts are needed to confirm these results and establish their translational relevance.

## Data Availability

The data presented in the study are deposited in the NCBI repository, accession number PRJNA1283980.
