# Supplementary material for: Fecal microbiota transplantation augments 5-fluorouracil efficacy in pancreatic cancer via gut microbiota modulation
Source: Front Microbiol. 2025 Sep 25;16:1548027. doi: 10.3389/fmicb.2025.1548027 (PMC12507756; doi:10.3389/fmicb.2025.1548027)
Supplement: Supplementary file 1 [file Supplementary_file_1.zip › Supplementary materials/Surviving numbers .docx]

The survived numbers over ten weeks among five groups

| Week | Sham | Model | FMT | 5FU | FMT + 5FU |
| --- | --- | --- | --- | --- | --- |
| 0 | 20 | 20 | 20 | 20 | 20 |
| 1 | 20 | 20 | 20 | 20 | 20 |
| 2 | 20 | 18 | 20 | 20 | 20 |
| 3 | 20 | 16 | 19 | 18 | 20 |
| 4 | 17 | 16 | 18 | 17 | 18 |
| 5 | 17 | 13 | 14 | 14 | 18 |
| 6 | 17 | 10 | 17 | 13 | 14 |
| 7 | 13 | 8 | 11 | 11 | 14 |
| 8 | 13 | 7 | 9 | 8 | 9 |
| 9 | 13 | 5 | 9 | 7 | 9 |
| 10 | 10 | 3 | 8 | 6 | 8 |

Note: Here’s a summary table showing, out of n = 20 mice per group, how many remain alive at each weekly time-point (Week 0 = baseline through Week 10)
