# Supplementary material for: Fecal microbiota transplantation augments 5-fluorouracil efficacy in pancreatic cancer via gut microbiota modulation
Source: Front Microbiol. 2025 Sep 25;16:1548027. doi: 10.3389/fmicb.2025.1548027 (PMC12507756; doi:10.3389/fmicb.2025.1548027)
Supplement: Supplementary file 1 [file Supplementary_file_1.zip › Supplementary materials/WB-wholegelcontrol.pptx]

## Slide 1
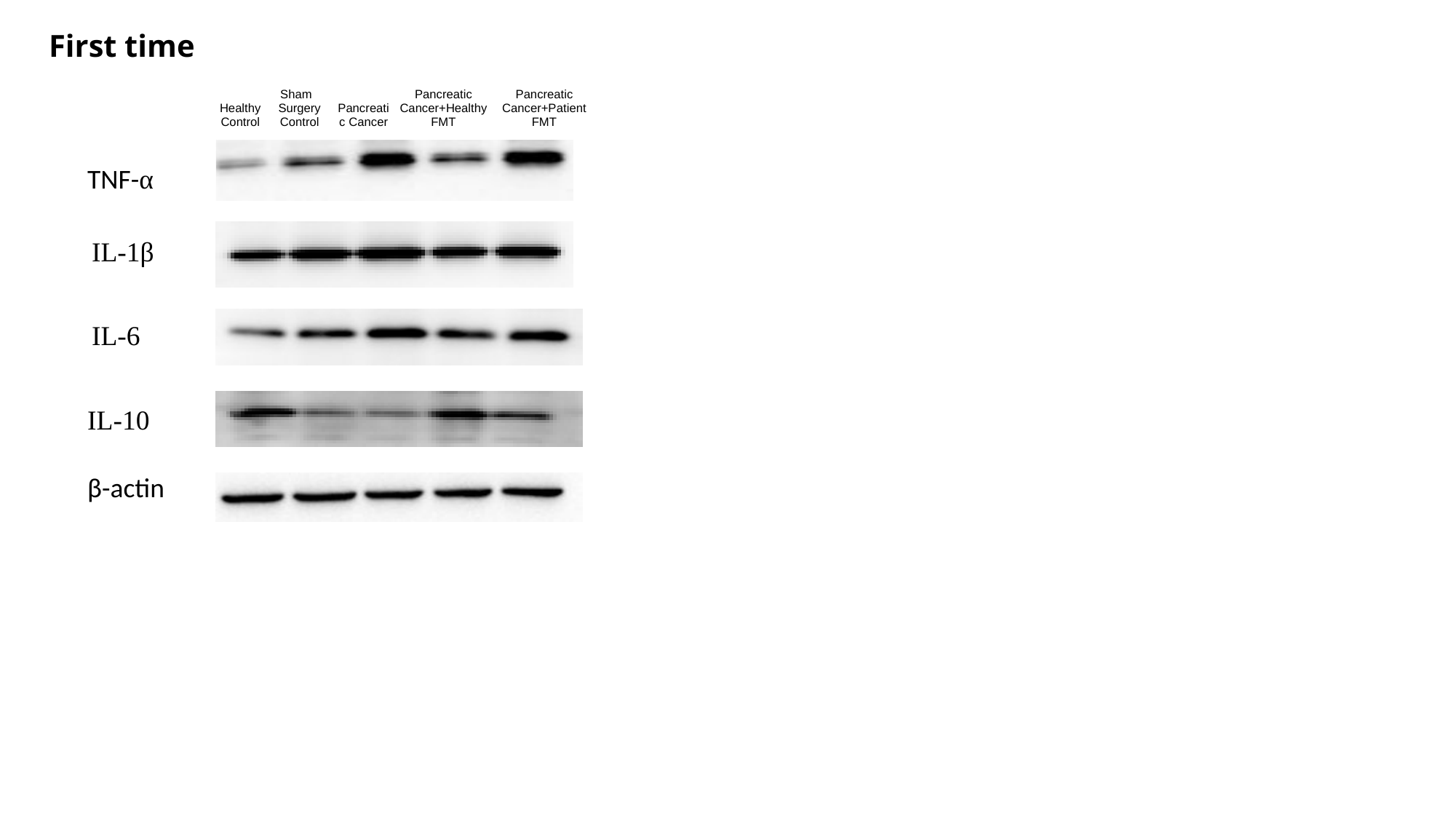

First time
| Healthy Control | Sham Surgery Control | Pancreatic Cancer | Pancreatic Cancer+Healthy FMT | Pancreatic Cancer+Patient FMT |
| --- | --- | --- | --- | --- |
TNF-α
IL-1β
IL-6
β-actin
IL-10

## Slide 2
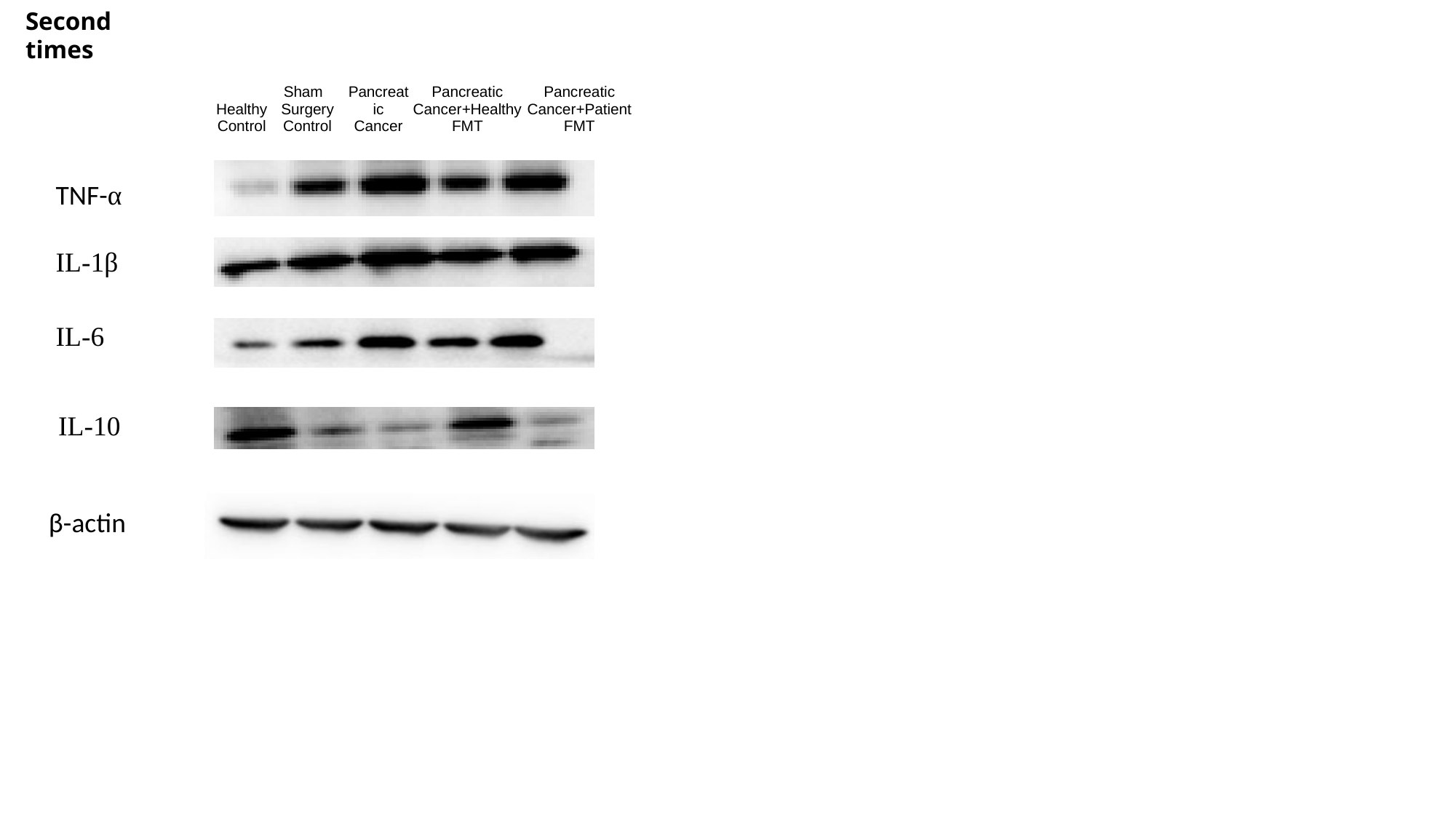

Second times
| Healthy Control | Sham Surgery Control | Pancreatic Cancer | Pancreatic Cancer+Healthy FMT | Pancreatic Cancer+Patient FMT |
| --- | --- | --- | --- | --- |
TNF-α
IL-1β
IL-6
IL-10
β-actin

## Slide 3
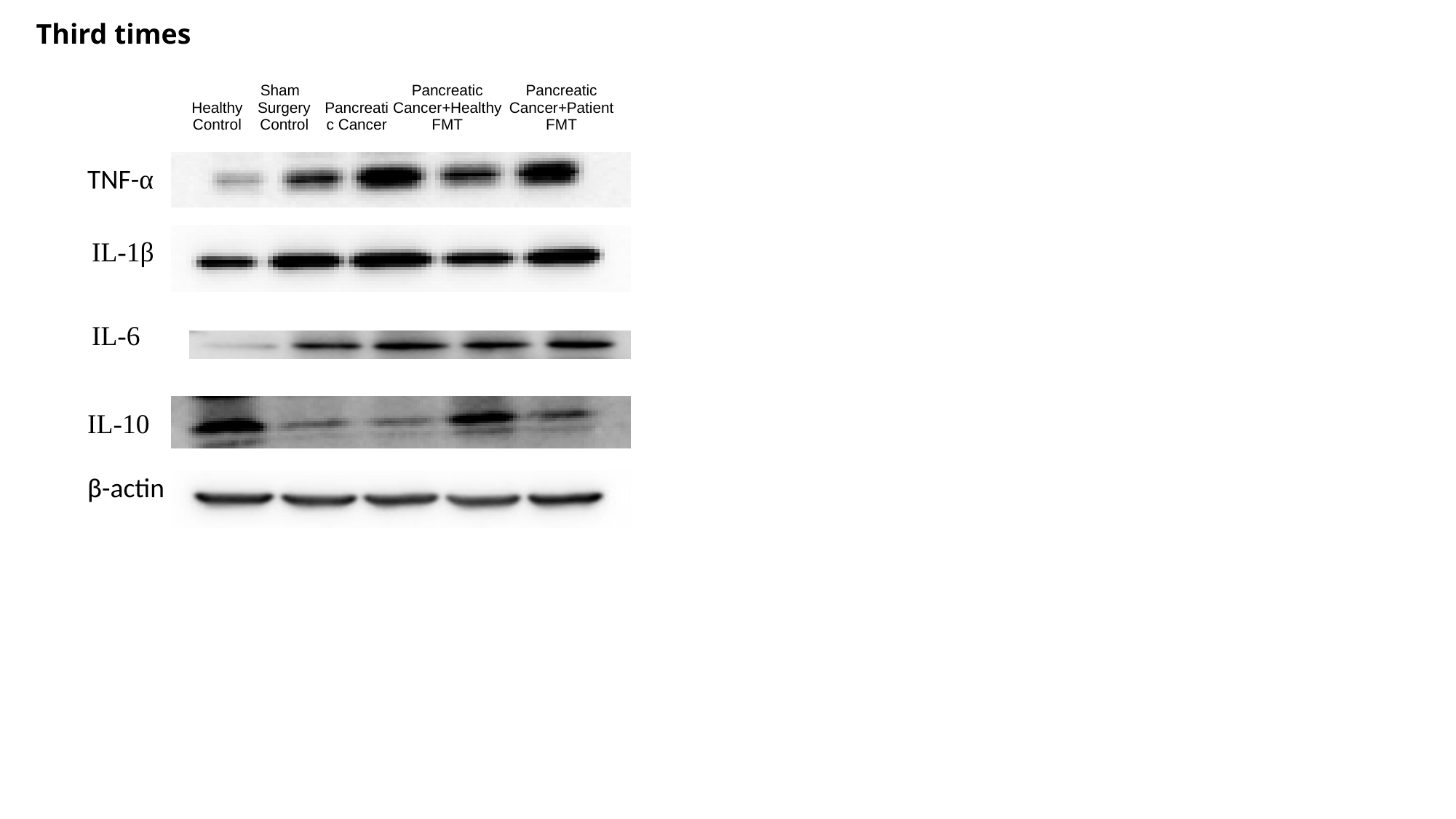

Third times
| Healthy Control | Sham Surgery Control | Pancreatic Cancer | Pancreatic Cancer+Healthy FMT | Pancreatic Cancer+Patient FMT |
| --- | --- | --- | --- | --- |
TNF-α
IL-1β
IL-6
β-actin
IL-10

## Slide 4
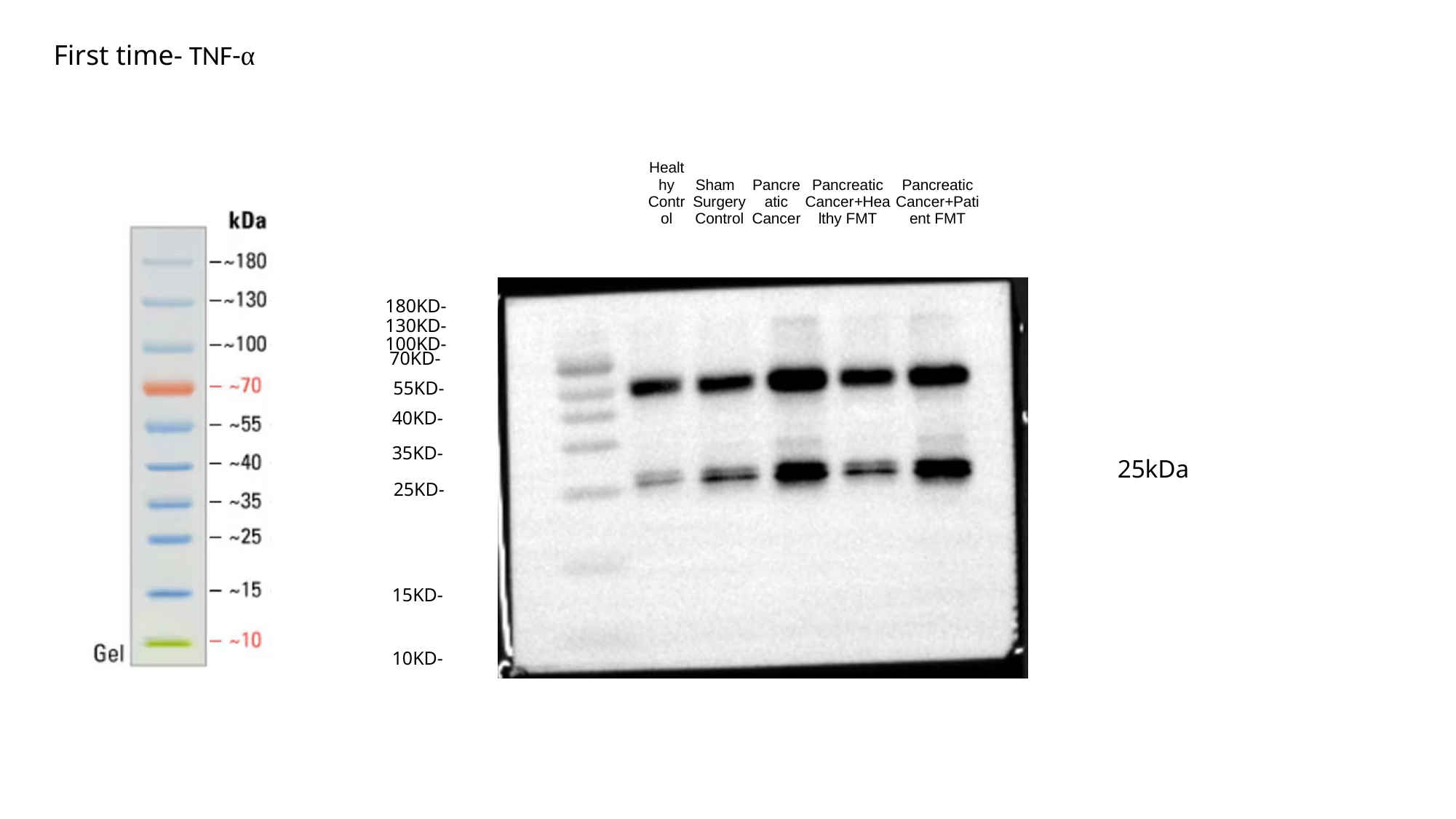

First time- TNF-α
| Healthy Control | Sham Surgery Control | Pancreatic Cancer | Pancreatic Cancer+Healthy FMT | Pancreatic Cancer+Patient FMT |
| --- | --- | --- | --- | --- |
180KD-
130KD-
70KD-
55KD-
40KD-
25kDa
10KD-
100KD-
35KD-
25KD-
15KD-

## Slide 5
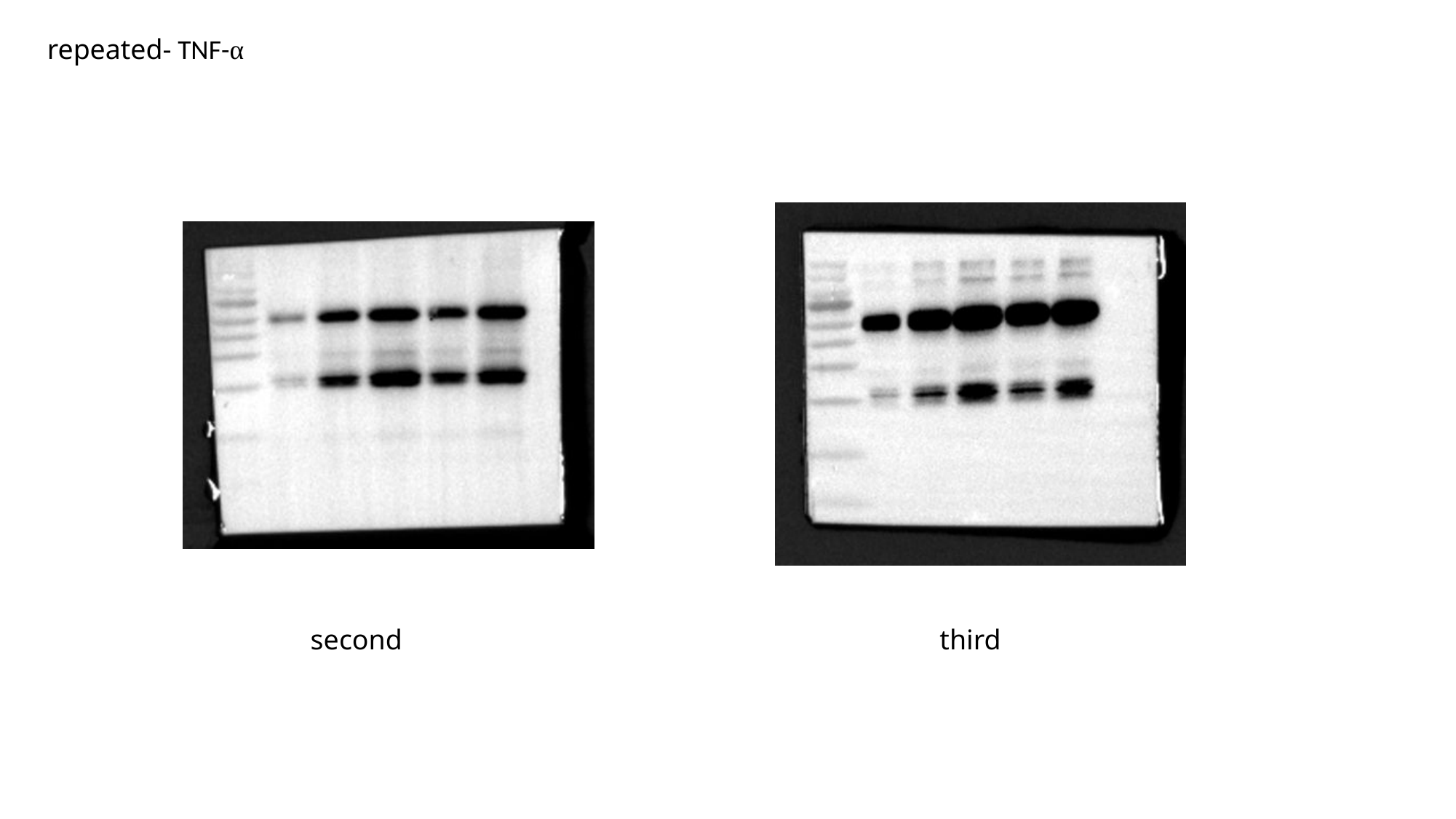

repeated- TNF-α
second
third

## Slide 6
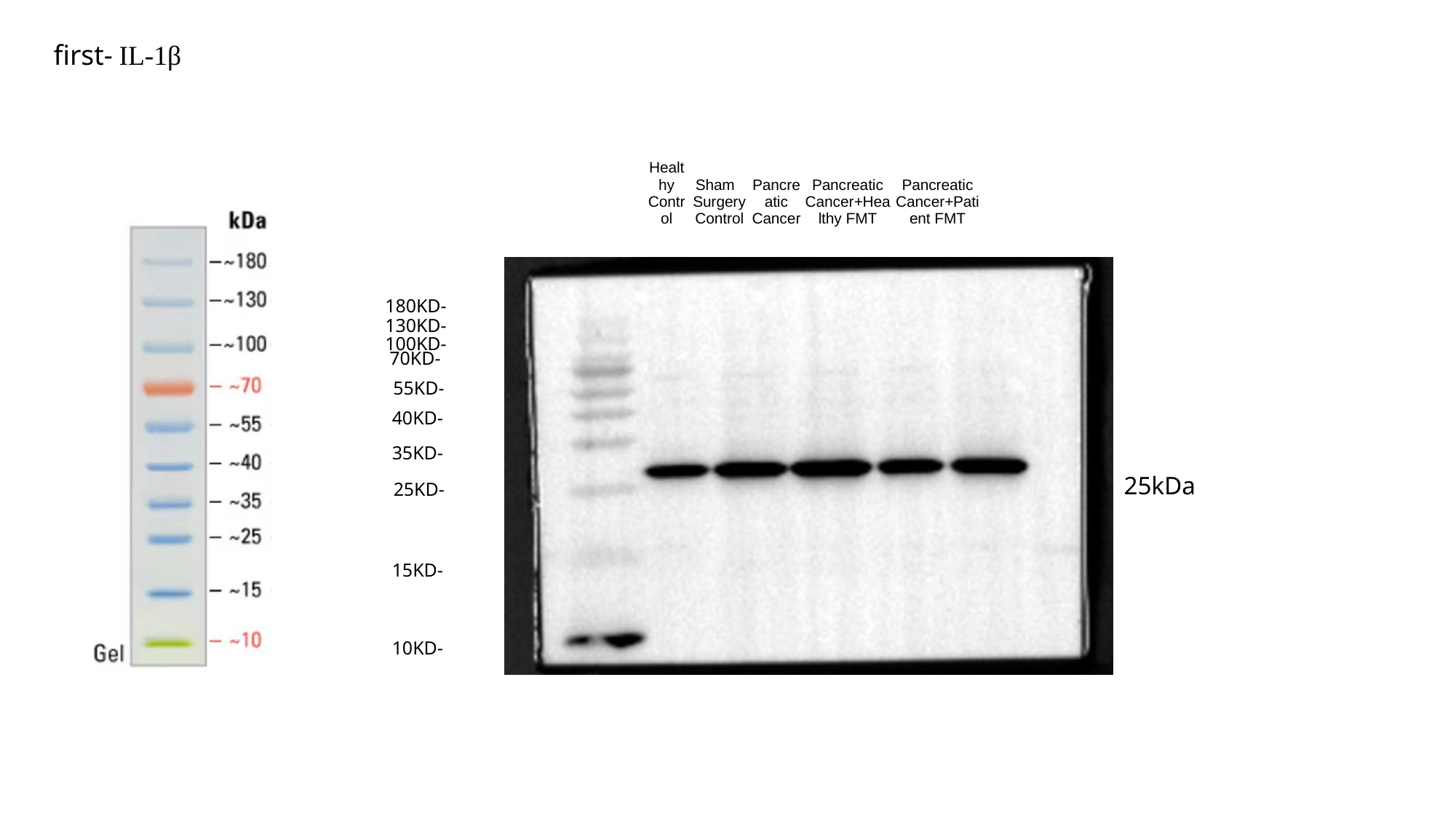

first- IL-1β
| Healthy Control | Sham Surgery Control | Pancreatic Cancer | Pancreatic Cancer+Healthy FMT | Pancreatic Cancer+Patient FMT |
| --- | --- | --- | --- | --- |
180KD-
130KD-
70KD-
55KD-
40KD-
25kDa
10KD-
100KD-
35KD-
25KD-
15KD-

## Slide 7
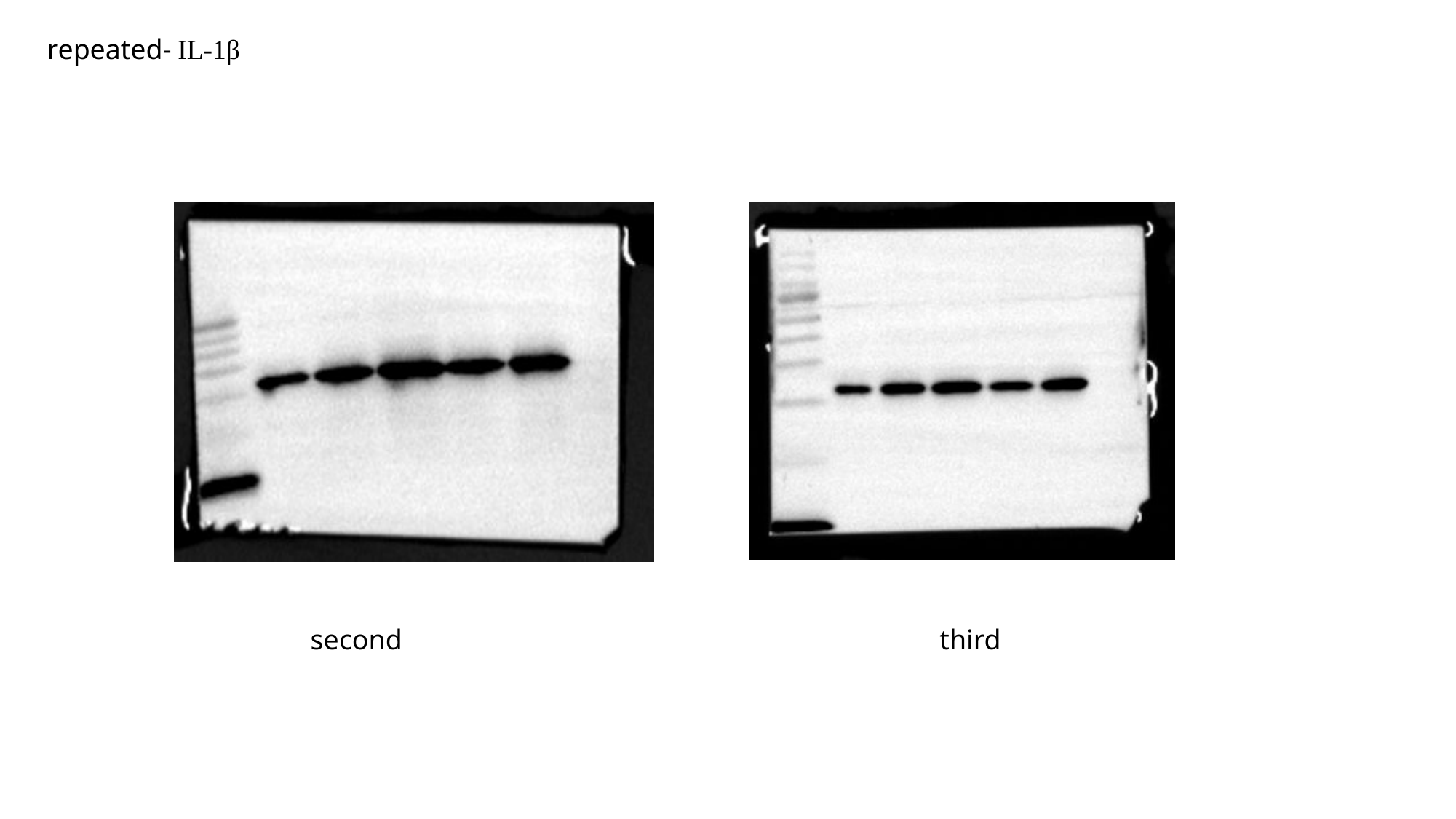

repeated- IL-1β
second
third

## Slide 8
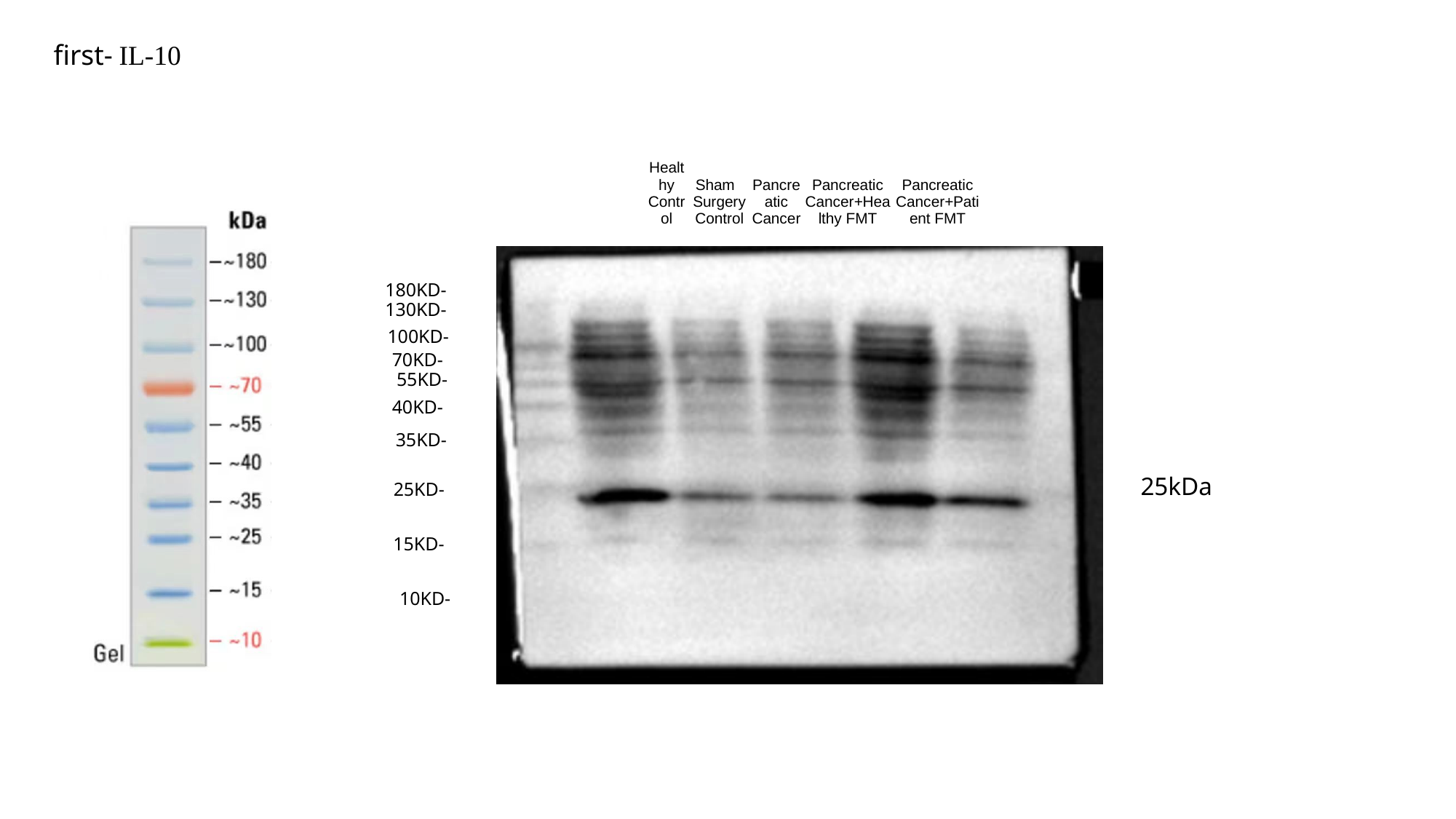

first- IL-10
| Healthy Control | Sham Surgery Control | Pancreatic Cancer | Pancreatic Cancer+Healthy FMT | Pancreatic Cancer+Patient FMT |
| --- | --- | --- | --- | --- |
180KD-
130KD-
70KD-
55KD-
40KD-
25kDa
10KD-
100KD-
35KD-
25KD-
15KD-

## Slide 9
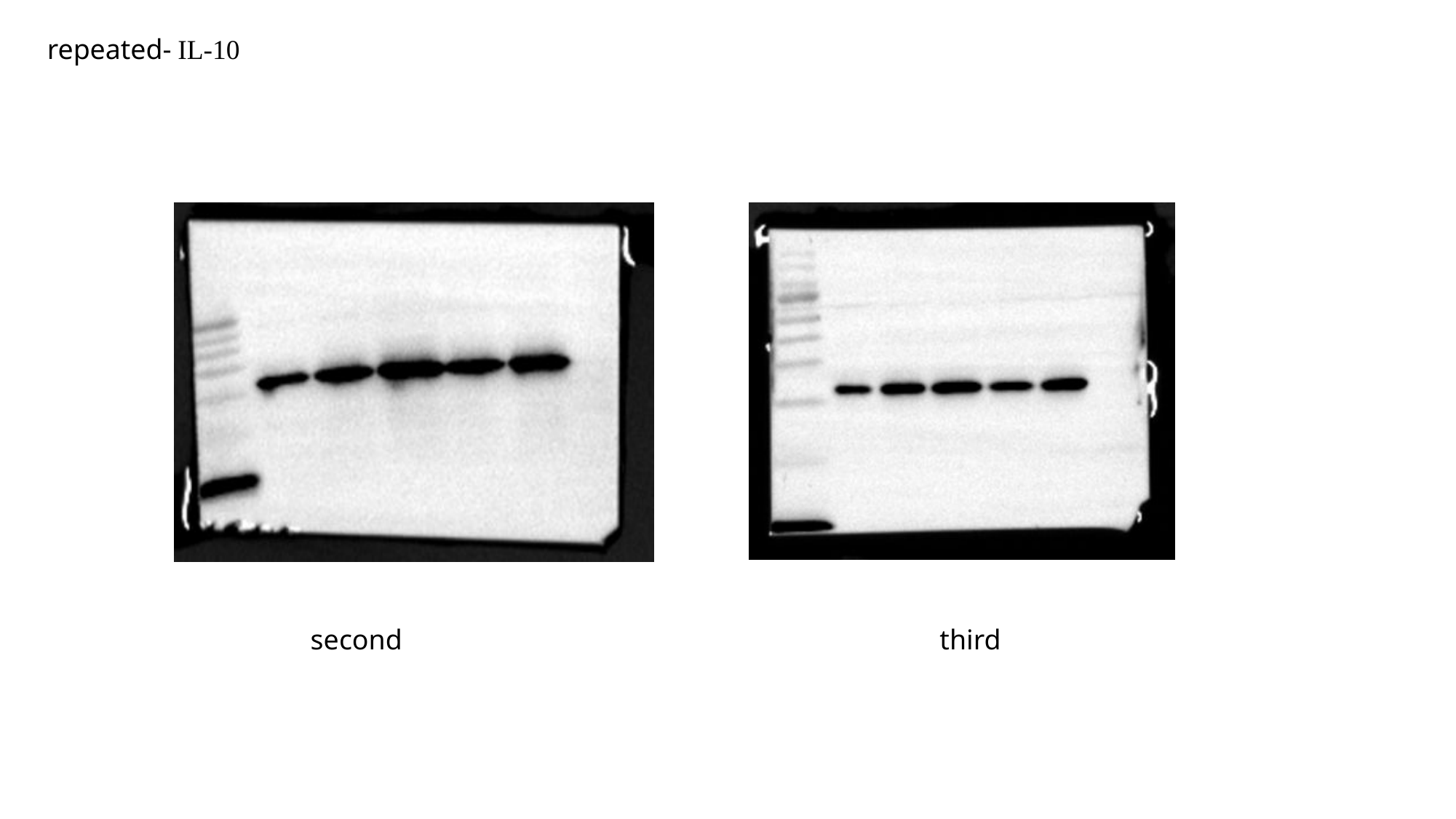

repeated- IL-10
second
third

## Slide 10
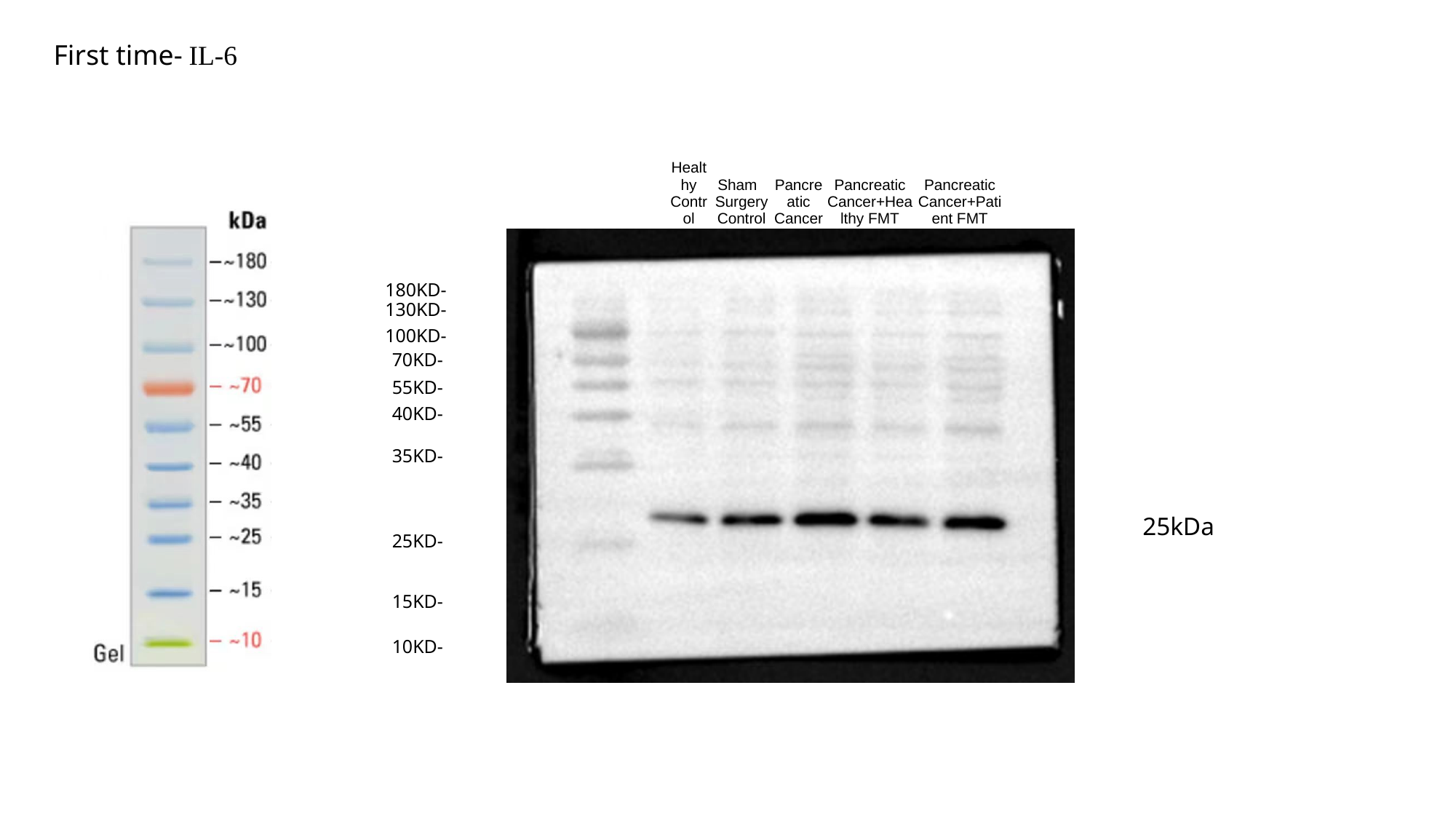

First time- IL-6
| Healthy Control | Sham Surgery Control | Pancreatic Cancer | Pancreatic Cancer+Healthy FMT | Pancreatic Cancer+Patient FMT |
| --- | --- | --- | --- | --- |
180KD-
130KD-
70KD-
55KD-
40KD-
25kDa
10KD-
100KD-
35KD-
25KD-
15KD-

## Slide 11
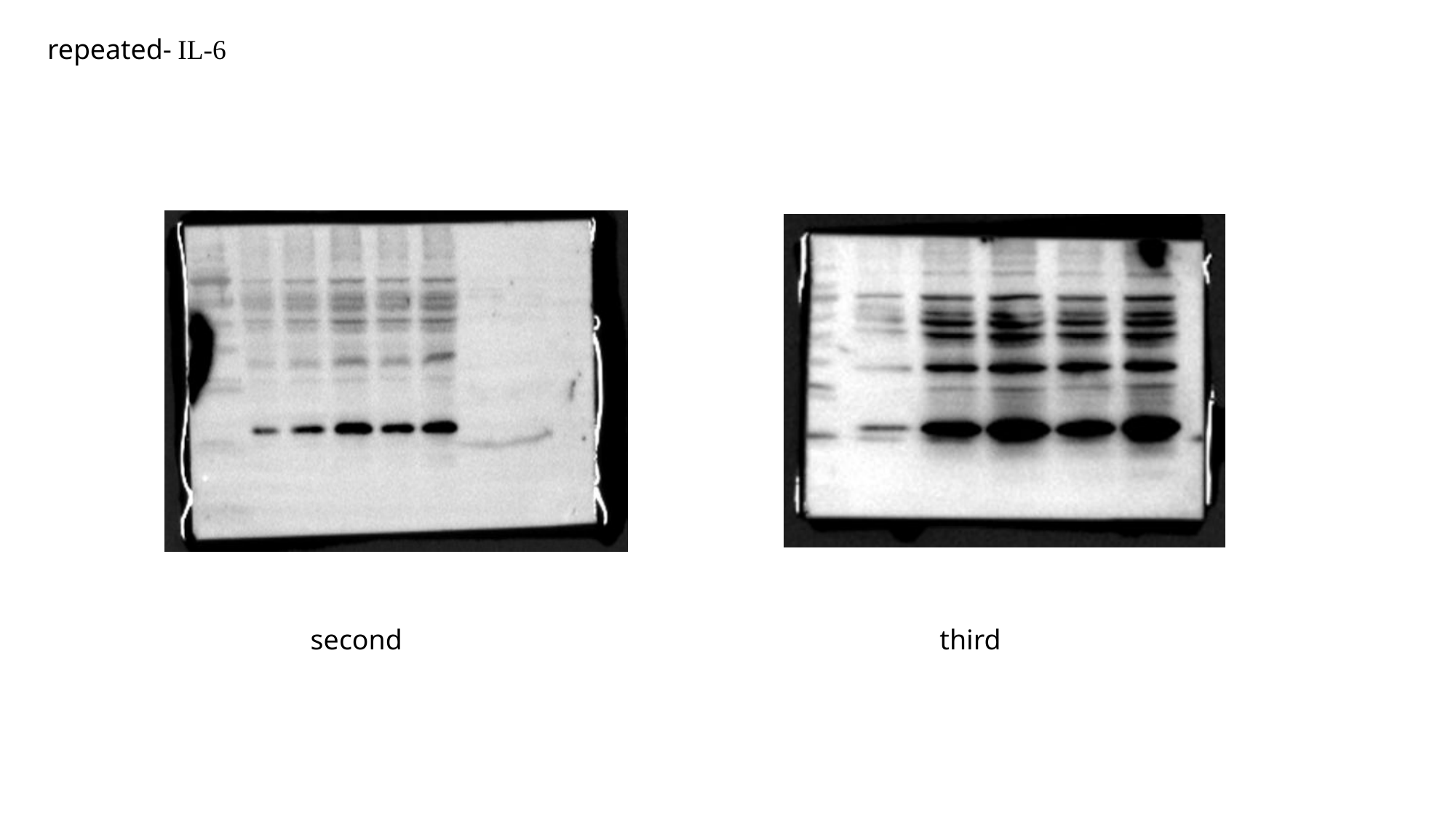

repeated- IL-6
second
third

## Slide 12
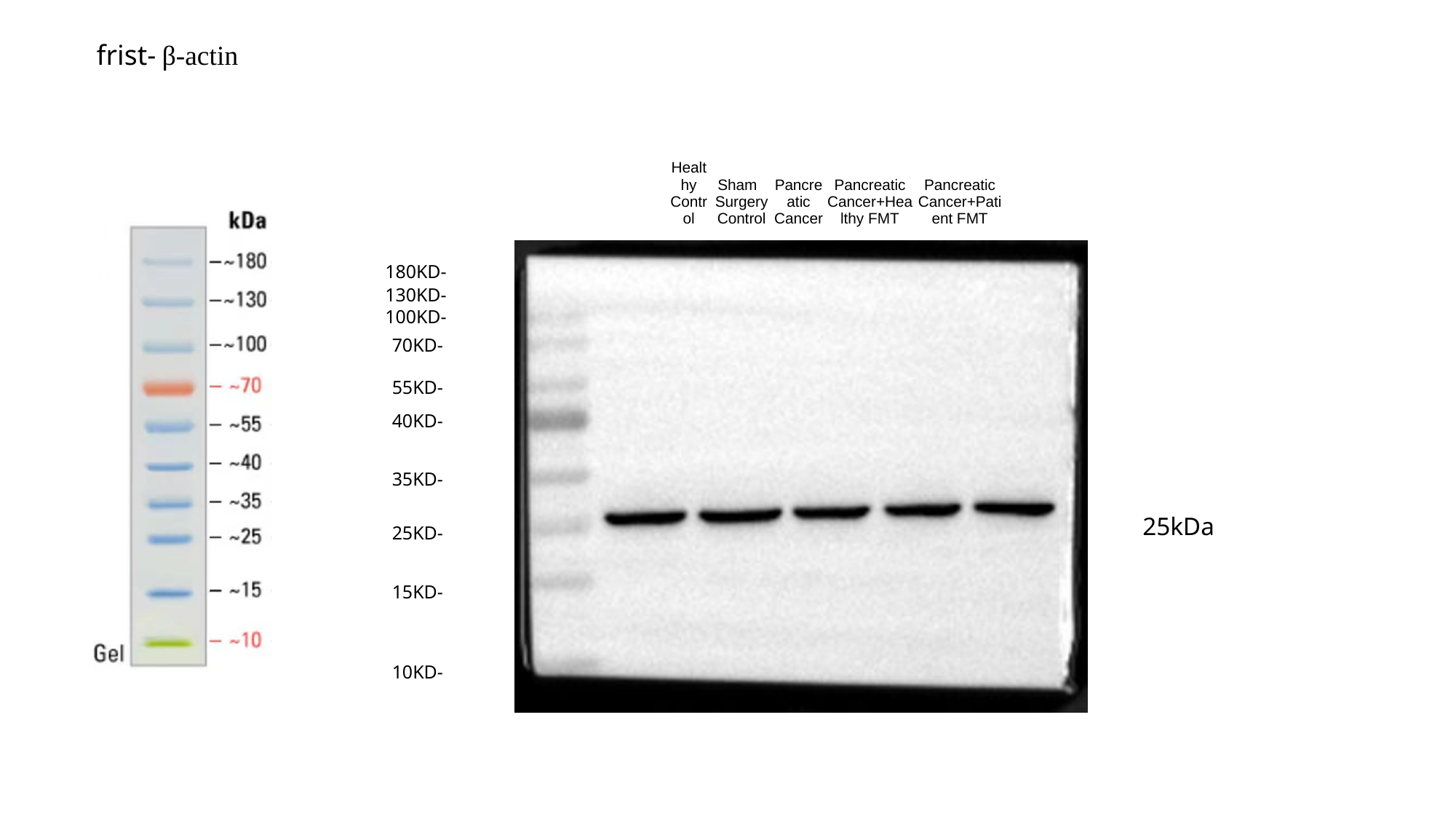

frist- β-actin
| Healthy Control | Sham Surgery Control | Pancreatic Cancer | Pancreatic Cancer+Healthy FMT | Pancreatic Cancer+Patient FMT |
| --- | --- | --- | --- | --- |
180KD-
130KD-
70KD-
55KD-
40KD-
25kDa
10KD-
100KD-
35KD-
25KD-
15KD-

## Slide 13
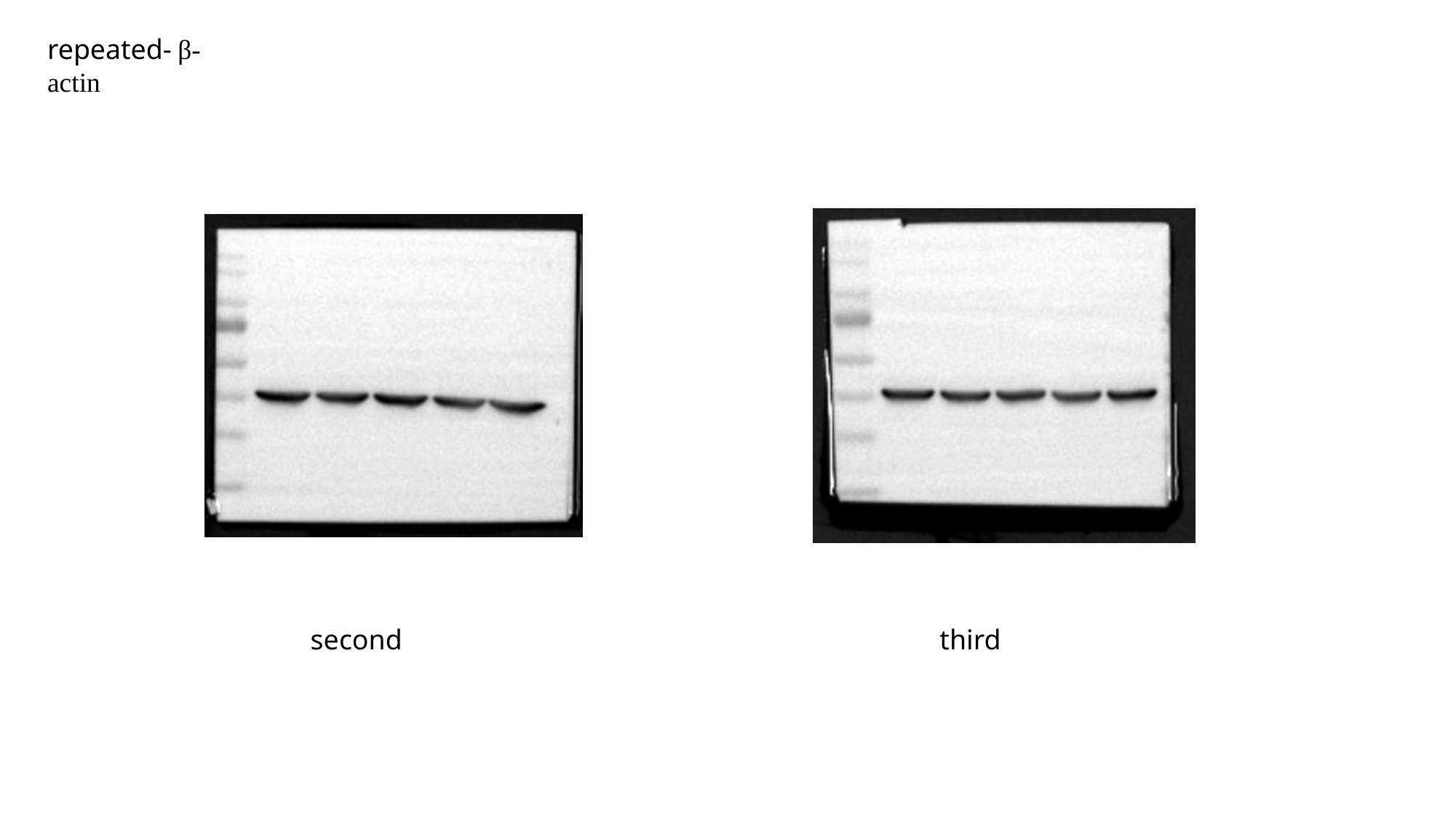

repeated- β-actin
second
third
