# Supplementary material for: Fecal microbiota transplantation augments 5-fluorouracil efficacy in pancreatic cancer via gut microbiota modulation
Source: Front Microbiol. 2025 Sep 25;16:1548027. doi: 10.3389/fmicb.2025.1548027 (PMC12507756; doi:10.3389/fmicb.2025.1548027)
Supplement: Supplementary file 1 [file Supplementary_file_1.zip › Supplementary materials/csv file legends.docx]

Fecal Microbiome ASV Abundance Tables for Five Experimental Groups

**Description:** Five separate CSV files—**Sham_Group_16S_ASV_Abundance.csv**, **Model_Group_16S_ASV_Abundance.csv**, **FMT_Group_16S_ASV_Abundance.csv**, **5FU_Group_16S_ASV_Abundance.csv**, and **FMT+5FU_Group_16S_ASV_Abundance.csv**—each contain a DADA2-generated ASV abundance matrix (raw read counts) derived from V3–V4 16S rRNA amplicon sequencing of fecal samples collected under sterile conditions. In each file, rows represent unique ASV identifiers and columns denote individual biological replicates. These counts reflect demultiplexing, quality filtering (Phred ≥ 30, truncated to 250 bp), chimera removal, pooled triplicate PCR cleanup, and denoising in QIIME 2. Taxonomic assignments based on the SILVA 138 99% OTU database are provided in an accompanying taxonomy export. These CSVs underpin downstream diversity analyses (α and β metrics), PICRUSt2 functional profiling, and differential-abundance testing (ANOVA/Tukey’s HSD, LEfSe).
